# Beyond the surface: accounting for confounders in understanding the link between collectivism and COVID-19 pandemic in the United States

**DOI:** 10.1186/s12889-023-16384-2

**Published:** 2023-08-09

**Authors:** Mac Zewei Ma, Sylvia Xiaohua Chen

**Affiliations:** Department of Applied Social Sciences, The Hong Kong Polytechnic University, Hung Hom, Kowloon, Hong Kong SAR China

**Keywords:** COVID-19, Collectivism, Behavioral immune system theory, Parasite-stress theory, Multilevel analysis, Covariates

## Abstract

According to the parasite-stress theory, collectivism serves as a trait of ingroup assortative sociality, providing defense against infectious diseases. This study investigated the association between cultural collectivism and COVID-19 severity at the state (Study 1: *N* = 51), county (Study 2: *N* = 3,133), and daily (Study 3: *N* = 52,806) levels from the beginning of 2020 to the end of 2022. State-level collectivism was assessed using two distinct measures: the U.S. collectivism index, focusing on social interconnectedness and interdependence, and the subjective-culture individualism-collectivism index (reversed), capturing attitudes and beliefs related to religion, abortion, and same-sex marriage. By employing random-intercept multilevel models, the results demonstrated significant and negative effects of state-level collectivism, as measured by the U.S collectivism index, on COVID-19 cases per million, COVID-19 deaths per million, and composite COVID-19 severity index, after controlling for confounding factors, such as socioeconomic development, ecological threats, disease protective behaviors, cultural norms, and political influences. A mini meta-analysis (Study 4: *N* = 9) confirmed the significance of these effects across studies. These findings supported the proactive role of collectivism in defending against the novel coronavirus in the United States, aligning with the parasite-stress theory of sociality. However, the subjective-culture individualism-collectivism index (reversed) did not exhibit a significant relationship with COVID-19 severity when confounding factors were considered. The high correlation between the subjective-culture individualism-collectivism index (reversed) and the controlled variables suggested shared variance that could diminish its impact on COVID-19 outcomes. Accordingly, the present findings underscore the significance of accounting for confounding factors when examining the association between collectivism and COVID-19 severity at population level. By considering relevant confounding factors, researchers could gain a comprehensive understanding of the complex interplay between cultural collectivism and its influence on COVID-19 severity. Overall, this research contributes to our understanding of how cultural collectivism shapes the COVID-19 pandemic in the United States, emphasizing the importance of adjusting for confounding effects in population level studies.

**Supplementary Information:**

The online version contains supplementary material available at 10.1186/s12889-023-16384-2.

## Introduction

Throughout history, infectious diseases have been a significant cause of human morbidity and mortality [1]. In response to these threats, humans have developed the behavioral immune system [2], a complex set of mechanisms encompassing emotional, cognitive, and behavioral responses aimed at defending against infectious diseases. Within this system, two primary types of responses have emerged: reactive and proactive responses [3, 4].

Reactive responses are activated in immediate perceptual environments where pathogen cues are salient [4]. Examples of such cues include detecting unpleasant odors associated with bacterial threats [5], encountering objects that are perceived as dirty and potentially disease-ridden [6], viewing images depicting scenes with high pathogen risk [7], or reading press reports about infectious disease threats [8].

In contrast, proactive responses are proposed to be a result of the powerful selection pressures imposed by infectious diseases on humans [4]. These responses are believed to be cultural values that have emerged as long-term strategies to avoid and manage the threats posed by infectious diseases [3, 9]. For instance, collectivistic values, which emphasize tradition, conformity, ingroup favoritism, and outgroup avoidance [10, 11], manifest as proactive disease-avoidance strategies [11].

The importance of social cohesion and cooperation within a group is emphasized through ingroup favoritism and outgroup avoidance, which are key features of collectivist values [12]. In the context of disease avoidance, collectivistic cultures may have developed cultural practices and norms that prioritize the health of the group over individual needs [11, 13, 14]. This can facilitate the sharing of health information and resources within the group and promote adherence to health guidelines and regulations by placing a higher value on conforming to social norms and following authority figures and institutions [15, 16].

Furthermore, it is suggested that collectivistic cultures have evolved proactive responses to the threat of infectious diseases. Studies indicate that collectivistic cultures are more likely to adopt cultural practices that help reduce disease transmission [11, 17, 18]. These practices may include establishing taboos against consuming certain foods, adhering to norms regarding personal hygiene, and implementing rituals related to illness and death [13, 19–21]. Over time, these practices have likely evolved as effective means to limit the spread of infectious diseases within the group, thereby increasing the group’s chances of survival.

Accordingly, the parasite-stress theory of sociality posits that collectivism is an adaptive human response to elevated parasite-stress [11, 17, 18]. Empirical evidence strongly supports this theory, with studies consistently demonstrating a relationship between pathogen prevalence and collectivism and its fundamental components [11, 22–24].

One multilevel study provides compelling evidence by showing that pathogen prevalence significantly predicts conservative political ideology [24], a finding later corroborated by other researchers [25, 26]. In countries with higher pathogen prevalence, parents also tend to socialize their children to value obedience as a protective measure [27]. Furthermore, analysis of U.S. data shows that state-level pathogen prevalence positively predict implicit and explicit racism and racial prejudice in both African and European Americans [25].

Experimental research has further shed light on the relationship between pathogen threats and ingroup favoritism and outgroup avoidance. Exposure to pathogen threats has been shown to induce greater levels of ethnocentrism [28], xenophobia [29] and ingroup conformity [30]. These findings suggest that humans have evolved to respond to the threat of infectious diseases by developing collectivistic values and behaviors, ultimately enhancing the survival prospects of the group.

The substantial empirical evidence supporting the parasite-stress theory underscores the profound influence of infectious diseases on human social behavior. It illuminates the adaptive nature of collectivism as a response to the threats posed by pathogens and underscores the pivotal role of diseases in shaping cultural values, political ideologies, intergroup dynamics, and socialization practices. Given the premise that local cultural values, including collectivism, have evolved to safeguard populations against infectious diseases and optimize inclusive fitness, it is reasonable to hypothesize that regions characterized by high levels of collectivism would exhibit lower rates of COVID-19 infection and mortality.

This intuitive proposition finds support in the behavioral immune system theory and the parasite-stress theory of sociality. Numerous studies have demonstrated that population-level collectivism serves as a significant predictor of COVID-19 infection and mortality rates across different countries [31–36]. As postulated by the parasite-stress theory of sociality, which suggests a positive association between higher pathogen prevalence and stronger ingroup assortative sociality [11, 13, 14, 17, 23], findings at the group level have further indicated that ingroup assortative sociality is a proactive response to the COVID-19 pandemic [37, 38].

Indeed, strong ingroup assortative sociality is a key component of the behavioral immune system [38, 39]. High level of collectivism could be seen as a robust group-level proactive response of the behavioral immune system, attributable to the disease-avoidance and disease-management behaviors promoted by this cultural value.

This argument gains further support from empirical evidence indicating that regions characterized by stronger collectivistic cultural values have exhibited swifter mobilization of social distancing measures and more prompt implementation of government responses to COVID-19 [40, 41]. Additionally, at the individual level, higher levels of collectivism have been associated with increased worries and concerns about COVID-19, as well as greater intentions to adopt preventive measures [42–44].

If collectivism is indeed an evolutionarily adaptive value for avoiding and managing infectious diseases [39], it is reasonable to expect it to predict individual-level processes that guard against the novel coronavirus. Indeed, one multilevel analysis demonstrates that group-level collectivism positively and significantly predicted individual-level mask usage, even when accounting for various other factors [45]. These findings offer further support for the hypothesis that collectivistic cultural values have played a pivotal role in defending against the transmission of infectious diseases, including COVID-19.

Accordingly, the collective body of evidence strongly suggests that promoting collectivism may serve as an effective strategy to mitigate the COVID-19 pandemic, prompting researchers to investigate the efficacy of emphasizing collectivistic values during this crisis [46]. While it is widely acknowledged that higher levels of collectivism are associated with lower COVID-19 infection and mortality rates on a global scale [31, 32, 34–36], state-level analyses in the United States have yielded contradictory findings, indicating that higher levels of collectivism were positively and significantly linked to COVID-19 confirmed cases and deaths [36].

This intriguing contradiction warrants further investigation, as heightened ingroup assortative sociality is known to foster conformity and obedience [10, 11], making it plausible that individuals exhibiting collectivistic cultural traits would be more inclined to comply with government-mandated policies and regulations aimed at curbing the spread of the infection [45, 47–49]. This suggests that a high level of collectivism in the United States, as in other countries, should theoretically be associated with lower COVID-19 severity, given that collectivism fosters adherence to COVID-19 preventive measures in the United States [45, 50].

The apparent inconsistency between cross-national findings and state-level analyses regarding the connection between collectivism and COVID-19 severity may stem from several factors that warrant careful consideration. It is important to recognize that state-level analyses concentrate specifically on the United States, a nation with unique contextual dynamics that can influence the relationship. In essence, the interplay between cultural dimensions, such as collectivism, and other contextual factors may exhibit different patterns across nations and subnational regions.

Given the intricate nature of the association between collectivism and COVID-19 severity, it is susceptible to the influence of various confounding factors. Cross-national studies typically encompass a wide array of variables, including healthcare infrastructure, economic indicators, political systems, and cultural dimensions [37, 51, 52], which may moderate or mediate the relationship between collectivism and COVID-19 outcomes. On the other hand, state-level analyses, while offering a more localized perspective [53], may not fully account for all confounding variables, potentially leading to disparate findings.

Although recent studies have conducted group-level analyses to explore the relationship between collectivism and the COVID-19 pandemic [31–36], establishing a true causal relationship requires careful consideration of relevant confounding variables, especially when relying on observational data [54, 55]. Indeed, recent studies have shown that multiple factors could influence the severity of COVID-19 across regions [37, 38, 45, 51, 52, 56–59], suggesting the need to carefully consider specific factors that might confound the association.

Firstly, collectivist cultures often prioritize social cohesion and the well-being of the group, which could lead to higher compliance with preventive measures [45, 47–49]. Accounting for the effects of protective behaviors is crucial in revealing a clearer and more direct relationship between collectivism and COVID-19 severity. Moreover, collectivist cultures tend to exhibit high levels of trust in authority figures and institutions [15, 16]. This trust might translate into better adherence to guidelines and recommendations from health authorities like the Centers for Disease Control and Prevention, resulting in more effective disease prevention and control measures. Therefore, it is important to consider the impact of trust in health authority information when examining the effect of collectivism on COVID-19 severity.

Secondly, socioeconomic factors influence access to healthcare services, testing availability, and the quality of healthcare systems [60–64]. Therefore, incorporating socioeconomic factors such as personal income, education level, population density, and healthcare infrastructure [31, 32, 34–37, 45] could provide a comprehensive understanding of the relationship between collectivism and COVID-19 severity.

Thirdly, political factors can also significantly confound the relationship between collectivism and COVID-19, particularly in the United States. The observed political polarization in various domains, including public health issues, has implications for COVID-19 [65]. Within the context of the pandemic, political affiliation can influence the formation of social networks and the adoption of preventive behaviors [33, 65]. Indeed, individuals tend to conform to the prevailing behaviors and attitudes within their political affiliations [66–68], potentially leading to divergent patterns of COVID-19 severity [65]. Accordingly, considering the influence of political affiliation in the United States becomes crucial as it may confound the effect of collectivism on COVID-19 severity due to polarization effects on social dynamics [33, 45, 65, 69].

Fourthly, inclusion of additional variables such as the historical parasite-stress index, HIV infection rate, latitude, and social vulnerability index provides valuable insights into the influence of historical and environmental factors on COVID-19 severity. These variables capture factors such as previous exposure to infectious diseases, environmental conditions, and social vulnerability [57, 70, 71]. Controlling for the HIV infection rate is especially important when examining the role of collectivism in COVID-19 severity, as the prevalence of sexually transmitted diseases acts as a proxy for life history strategy at the population level [72], which predicts COVID-19 preventive behaviors [73, 74].

Fifthly, adjusting for the effect of cultural tightness-looseness is crucial when investigating the impact of collectivism on COVID-19 severity in the United States, given the potential confounding role of this cultural dimension [45]. Cultural tightness characterizes societies with strong norms, strict rules, and low tolerance for deviant behavior, while cultural looseness describes societies with weaker norms, more permissive attitudes, and higher tolerance for diversity [52, 75, 76]. Collectivism is often associated with stronger obedience and a greater emphasis on conformity and interdependence [19, 27, 45, 77, 78]. In this context, collectivist values may translate into strict adherence to rules and social norms in tight cultures, potentially contributing to more cautious and compliant behaviors, including greater adherence to public health measures. Conversely, in loose cultures, individual autonomy and personal freedom may be valued more, leading to weaker adherence to collective behaviors such as compliance with public health guidelines. Failing to account for the effect of cultural tightness and looseness could distort the relationship between collectivism and COVID-19 severity.

Additionally, adjusting for the effect of religiosity is also essential when investigating the relationship between collectivism and COVID-19 severity in the United States due to the potential confounding role of religious beliefs and practices [79]. Religious beliefs and practices can shape individuals’ attitudes, behaviors, and decision-making processes [80–83], including their response to public health measures and adherence to guidelines during the COVID-19 pandemic [84–88]. As religiosity often involves collective rituals, community gatherings, and shared religious practices [89–92], the concept of religiosity is closely tied to social norms and values within religious groups or communities. In this context, collectivist values may be reinforced by religious teachings, promoting solidarity, cooperation, and shared responsibility. This emphasis on collective values may influence individuals’ compliance with public health guidelines, as religious communities often prioritize protecting the health and well-being of their members [13, 37]. Thus, failing to account for the effect of religiosity may lead to a misinterpretation of the relationship between collectivism and COVID-19 severity.

In the current research, a series of four comprehensive studies were conducted to thoroughly examine the association between collectivism and COVID-19 severity in the United States, building upon the disease-avoidant role of collectivism proposed by the influential parasite-stress theory. The primary objective of this research is to provide empirical support for the ecological validity of the parasite-stress theory and the proactive response of cultural collectivism within the behavioral immune system at a population level, while meticulously accounting for a range of potential confounding variables. The integration of these studies is poised to yield invaluable insights that can significantly inform policymaking decisions at the population level, equipping policymakers with the necessary knowledge to navigate the complexities of managing COVID-19 [93, 94].

Study 1 focuses on the analysis of state-level data to unveil a general pattern elucidating the correlation between collectivism and COVID-19 severity while meticulously adjusting for the effects of covariates. In Study 2, an investigation is conducted to examine the impact of state-level collectivism on predicting county-level COVID-19 severity, incorporating the control of covariates at both the state and county levels. Building upon these insights, Study 3 delves further into the effect of state-level collectivism on daily-level COVID-19 severity, employing rigorous controls for covariates at both the daily and state levels. To consolidate the findings and enhance their generalizability, Study 4 will conduct a mini meta-analysis to rigorously assessing the significance of the overall effect of collectivism across the integrated studies.

By meticulously conducting these interrelated studies, this research sets the stage for a deeper understanding of the relationship between collectivism and COVID-19 severity in the United States. The implications of these findings extend beyond academia and have significant practical implications for policymakers and public health professionals. It equips them with evidence-based insights and robust empirical evidence to inform decision-making processes, contributing to more effective strategies in the battle against the spread of COVID-19.

## Study 1

Study 1 sought to investigate the impact of state-level collectivism on state-level COVID-19 severity while accounting for potential confounding factors. To achieve this, a comprehensive set of relevant covariates was incorporated into the study design. The research data and code can be accessed through the Open Science Framework (OSF) repository at the following link: https://osf.io/436zm/?view_only=5173a1bb043648afa76621d218e6f75c. For additional details regarding the timeframes and sources of the original data, please refer to Appendix 1 in the Supplementary File.

### Method

#### States and timeframe

The analyses included data from all 50 states and Washington D.C. of the United States. The epidemiological data encompassed the period from the initial documentation of the first COVID-19 confirmed cases in each state to December 31, 2022. For the state-level analysis conducted in this study, the daily average value of each measure was calculated for every state. The specific timeframes for all variables can be found in Appendix 1 of the Supplementary File.

#### Measures

##### COVID-19 severity indicators

The present study utilized two commonly used indicators, COVID-19 cases per million and COVID-19 deaths per million, to assess the severity of the pandemic [32–36, 45, 52, 95–99]. Daily COVID-19 epidemiological data were obtained from the New York Times dataset, and the mean daily new confirmed cases (per million) and mean daily new confirmed deaths (per million) were calculated. To ensure normal distribution of the data, a log transformation was applied [36, 98–100].

##### Collectivism-individualism indices

###### U.S. collectivism index

Aligned with recent studies [36, 45], the present study adopted the U.S collectivism index [101] as a robust measurement tool. This index offers a comprehensive assessment of collectivism-individualism by incorporating a diverse set of objective indicators of cultural practices, thereby enhancing its ecological validity. The U.S collectivism index aggregates eight separate state-level indicators, sourced from highly representative data, ensuring its objectivity in measuring cultural collectivism in the United States.

The indicators utilized in the U.S collectivism index include the proportion of people living alone, the proportion of seniors living alone (reversed), the percentage of multi-generational households (reversed), the marriage to divorce ratio, the percentage of individuals with no religious affiliation (reversed), the percentage of people carpooling to work, the percentage of voters supporting the Libertarian party (reversed), and the percentage of self-employed workers (reversed). By incorporating these indicators, The U.S collectivism index captures the multifaceted dimensions of collectivism at the state level.

The creators of this index, in their extensive research, unveiled distinct geographic variations in collectivism among the 50 U.S. states [101], providing compelling evidence for cultural diversity within the nation. Moreover, their study revealed substantial associations between state-level collectivism scores and a range of crucial economic, social, and health outcomes. Notable correlations were observed between collectivism scores and indicators such as self-run farms (*r* = –.59), percentage of minority population (*r* = .75), gender equality (*r* = –.45), suicide rates (*r* = –.31), and rates of alcohol abuse (*r* = –.28). Consequently, accounting for sociocultural confounders became imperative in examining the relationship between the U.S collectivism index and COVID-19 severity.

###### Subjective-culture individualism-collectivism index (reversed)

The subjective-culture individualism-collectivism measure (reversed) [102] was used. It was derived by conducting a factor analysis on three variables: the percentage of highly religious individuals, the percentage opposing abortion, and the percentage opposing same-sex marriage. To obtain the final score for each U.S. state, the factor scores were multiplied by − 100. Thus, a higher score indicated a greater subjective individualistic orientation. In the current study, the original subjective-culture individualism-collectivism index was multiplied by − 1, resulting in higher scores indicating a greater subjective collectivistic orientation (i.e., subjective-culture individualism-collectivism (reversed)).

The original subjective-culture individualism-collectivism measure showed a small correlation with the U.S. collectivism index (*r* = –.24), indicating that they were related but distinct constructs. As previously mentioned, the U.S. collectivism index incorporated several objective indicators of collectivistic practices, while the subjective-culture individualism-collectivism index was based on three subjective measures of individualistic-collectivistic orientation. Therefore, examining the predictive effects of both the U.S collectivism index and the subjective-culture individualism-collectivism index on COVID-19 severity would provide a more comprehensive understanding of the role of collectivism during the pandemic in the United States.

The subjective-culture individualism-collectivism index exhibited strong associations with various socio-political-cultural factors [102], including median household income (*r* = .67), GDP per capita (*r* = .54), the percentage who voted for Trump in 2016 (*r* = − .77), the percentage who voted for Trump in 2020 (*r* = − .79), and the cultural tightness-looseness index (*r* = − .87). Therefore, when examining the relationship between this index and COVID-19 severity, it was crucial to control for these socio-political-cultural factors in order to isolate the unique contribution of collectivism to the understanding of the COVID-19 pandemic.

##### Covariates

In the present study, various covariates were carefully controlled for to accurately investigate the relationship between collectivism and COVID-19 severity in the United States.

###### Proxies of COVID-19-related prevention behaviors

The present study controlled for several proxies of COVID-19-related prevention, including stay-at-home behaviors, the COVID-19 stringency index, the percentage of people tested for COVID-19 in the past 14 days (regardless of their test results), the percentage of people wearing masks in the last 5 days, and the percentage of people who trust the Centers for Disease Control (CDC) to provide accurate news and information about COVID-19. Additionally, to account for the impact of anxiety on individuals’ behavior during the pandemic [103], the percentage of people reporting feeling anxious about the virus in the past 5 days was included. These variables were derived from the Delphi US COVID-19 Trends and Impact Survey (CTIS) conducted in partnership with Facebook. The survey collects data from tens of thousands of Facebook users daily and includes a comprehensive range of COVID-related questions, including inquiries about COVID-related symptoms experienced by individuals or their household members. For the current study, the daily average scores of these variables were calculated for each state from September 8, 2020, to March 15, 2021.

Furthermore, stay-at-home behavior was assessed using Google’s COVID-19 Community Mobility Reports, which provide data on the time people spent at different location categories such as retail & recreation, grocery & pharmacy, parks, transit stations, workplace, and residential (https://www.google.com/covid19/mobility/). Previous research demonstrated a strong negative relationship between residential movement and other types of movement [104]. To capture non-residential movement on a daily basis, this study calculated the average daily mobility scores for retail & recreation, grocery & pharmacy, parks, transit stations, and workplace, which were then subtracted from the daily mobility score of residential movement. This approach created an index reflecting the extent to which people practiced stay-at-home behavior on a daily basis [105]. A daily average value was calculated for each state, spanning from February 15, 2020, to October 15, 2022.

Additionally, the COVID-19 Stringency Index, developed by the University of Oxford, was used to assess policy strictness using 19 indicators [106]. The Oxford COVID-19 Government Response Tracker (OxCGRT) was utilized to obtain daily time series data on the stringency index for each U.S. state. Subsequently, the daily average score was calculated for each state, covering the period from January 1, 2020, to December 31, 2022.

###### Socioeconomic-political factors

Personal income per capita (log-transformed), population density (log-transformed), median age, male percentage, education level, social vulnerability, airport traffic, percentage of non-white population, and political affiliation (measured by the state-level voting gap in the 2016 election, where a higher score indicated a higher level of conservative political ideology) were considered in this study. These factors have been found to be associated with COVID-19 morbidity and mortality rates, as well as COVID-19-related preventive behaviors in previous research [36, 38, 45, 58, 59, 65].

###### Ecological factors

Latitude, historical prevalence of infectious diseases (excluding sexually transmissible diseases), HIV infection rate, and life expectancy at birth were included as controlled variables in this study. The inclusion of latitude was based on previous findings that climatological factors play a role in the spread of COVID-19 [56]. The historical prevalence of infectious diseases, calculated from state-wise median infection-related mortality rates from 1979 to 1998 [72], was controlled to account for the direct impact of parasite-stress on future disease outbreaks [57]. The historical parasite-stress index was created without including the prevalence rate of human immunodeficiency virus (HIV) to differentiate the effect of fast life history strategy from parasite-stress, as HIV prevalence rate serves as a proxy for people’s engagement in risky behaviors associated with a fast life history strategy [72]. However, the HIV infection rate was included in the analysis to account for the influence of life-history strategy on COVID-19 [73, 74]. Additionally, life expectancy at birth reflects the survival prospects of a population at a given time, serving as an indicator of the cumulative threats that pose a risk to survival [76]. Therefore, state-level life expectancy was included as a reliable measure of aggregated ecological threats.

###### Cultural factors

To examine the specific impact of cultural collectivism on predicting COVID-19 severity, it was crucial to control for the influence of other cultural factors. Therefore, the effects of cultural tightness-looseness and religiosity were taken into consideration. Previous research has indicated a positive association between collectivism and both cultural tightness-looseness [75] and religiosity [38]. Furthermore, cultural tightness-looseness and religiosity have been found to be predictors of COVID-19 severity [37, 38, 52]. In this study, the revised cultural tightness-looseness index [107], which excluded the religiosity component in its calculation, was utilized. This approach allowed for isolating the unique contribution of cultural collectivism in predicting COVID-19 severity. Moreover, the most updated state-level religiosity index [38], which was created based on online big data, was utilized.

### Statistical approach

All analyses were conducted separately for COVID-19 cases per million and COVID-19 deaths per million. Moreover, to capture the average COVID-19 severity across states, these two indicators were *Z*-transformed to calculate a composite index, which was then subjected to a series of analyses.

First, a bivariate correlation analysis was performed to examine the simple correlations between collectivism and the COVID-19 severity indicators. Subsequently, a partial correlation analysis was conducted, controlling for the potential confounding effects of all covariates.

Next, an Ordinary Least Square (OLS) regression analysis was carried out. To address the issue of spatial autocorrelation, a multilevel analysis was performed [38, 108]. The states were categorized into nine distinct divisions based on the U.S. Census Bureau’s definitions, and a multilevel regression model was specified with the nine divisions as a random intercept. The suitability of the multilevel analysis was assessed using the Likelihood-ratio test (LRT) and Intraclass Correlation Coefficient (ICC) [51, 109].

The following equations describe the random-intercept multilevel model:

Level 1 (State-level):

Y_*ij*_ = B_0*j*_ + B_1_×Stay-at-home index_*ij*_ + B_2_×Stringenxcy index_*ij*_ + B_3_×Percent tested for COVID_*ij*_ + B_4_×Percent wearing mask_*ij*_ + B_5_×Percent trust CDC information_*ij*_ + B_6_×Percent feeling anxious_*ij*_ + B_7_×Latitude_*ij*_ + B_8_×Parasite-stress index_*ij*_ + B_9_×HIV infection rate_*ij*_ + B_10_×Life expectancy_*ij*_ + B_11_×Personal income_*ij*_ + B_12_×Population density_*ij*_ + B_13_×Percent male population_*ij*_ + B_14_×Median age_*ij*_ + B_15_×Percent college degree population_*ij*_ + B_16_×Social vulnerability index_*ij*_ + B_17_×Airport traffic_*ij*_ + B_18_×Percent non-white population_*ij*_ + B_19_×Partisanship_*ij*_ + B_20_×Religiosity index_*ij*_ + B_21_×Cultural tightness-looseness index_*ij*_ + B_22_×Subjective-culture individualism-collectivism (reversed) _*ij*_ + B_23_×U.S. collectivism index_*ij*_ + e_*ij*_

Level 2 (Division-level):

B_0*j*_ = γ_00_ + u_0*j*_.

where:

Y_*ij*_ represents the COVID-19 severity for state *i* in division *j*. B_0*j*_ represents the random intercept for division *j*, indicating the baseline COVID-19 severity for that division. B_1_ to B_23_ are the fixed-effect coefficients for the state-level predictors. γ_00_ represents the overall intercept for the division-level model. e_*ij*_ is the state-level residual term. u_0*j*_ is the division-level residual term. The model allows for the estimation of division-specific random intercepts (B_0*j*_) and the variation in those intercepts (u_0*j*_) while accounting for the effects of state-level predictors (B_1_ to B_23_) and the residual error (e_*ij*_).

### Results and discussion

The results revealed that the U.S collectivism index showed non-significant correlations with COVID-19 cases per million (*r* = .06, *p* = .672, 95% CI [–0.22, 0.33]) and COVID-19 deaths per million (*r* = .001, *p* = .997, 95% CI [–0.28, 0.28]). On the other hand, the subjective-culture individualism-collectivism index (reversed) demonstrated a significant relationship with COVID-19 cases per million (*r* = .37, *p* = .009, 95% CI [0.09, 0.58]) and COVID-19 deaths per million (*r* = .49, *p* < .001, 95% CI [0.24, 0.67]).

Furthermore, the U.S collectivism index exhibited a non-significant correlation with the composite COVID-19 severity index (*r* = .04, *p *= .802, 95% CI [–0.24, 0.31]), representing the average score across the *Z*-transformed COVID-19 cases per million and deaths per million. In contrast, the subjective-culture individualism-collectivism index (reversed) was significantly associated with the composite index (*r* = .50, *p* < .001, 95% CI [0.26, 0.68]).

However, when controlling for all covariates in a partial correlation analysis, the U.S collectivism index displayed significant negative correlations with COVID-19 cases per million (*r* = –.42, *p* = .022, 95% CI [–0.69, –0.05]), COVID-19 deaths per million (*r* = –.70, *p* < .001, 95% CI [–0.85, –0.43]), and the composite COVID-19 severity index (*r* = –.66, *p* < .001, 95% CI [–0.83, –0.38]). Conversely, the subjective-culture individualism-collectivism index (reversed) showed non-significant relationships with COVID-19 cases per million (*r* = –.01, *p* = .973, 95% CI [–0.39, 0.37]), COVID-19 deaths per million (*r* = –.07, *p* = .734, 95% CI [–0.44, 0.32]), and the composite index (*r* = –.05, *p* = .816, 95% CI [–0.42, 0.34]).

Next, an OLS regression analysis was conducted to further examine the relationships between collectivism indices and COVID-19 severity. The results are presented in Table S1. In Model 1, before controlling for covariates, the U.S collectivism index did not significantly predict COVID-19 cases per million (B = − 0.0001, *SE* = 0.001, β = –0.03, *t* = − 0.19, *p* = .849, 95% CI [–0.002, 0.001]), COVID-19 deaths per million (B = − 0.001, *SE* = 0.002, β = –0.12, *t* = − 0.93, *p* = .356, 95% CI [–0.005, 0.002]), or the composite index (B = − 0.01, *SE* = 0.01, β = –0.09, *t* = − 0.67, *p* = .504, 95% CI [–0.03, 0.01]). However, the subjective-culture individualism-collectivism index (reversed) showed a significant positive association with COVID-19 cases per million (B = 0.0002, *SE* = 0.0001, β = 0.37, *t* = 2.67, *p* = .010, 95% CI [0.0001, 0.0004]), COVID-19 deaths per million (B = 0.001, *SE* = 0.0002, β = 0.51, *t* = 3.96, *p* < .001, 95% CI [0.0003, 0.0011]), and the composite index (B = 0.004, *SE* = 0.001, β = 0.52, *t* = 4.05, *p* < .001, 95% CI [0.002, 0.007]).

However, when accounting for the effects of covariates, the U.S collectivism index exhibited a significant negative relationship with COVID-19 cases per million (B = − 0.003, *SE* = 0.001, β = –0.49, *t* = − 2.39, *p* = .025, 95% CI [–0.0050, − 0.0004]), COVID-19 deaths per million (B = − 0.01, *SE* = 0.002, β = –0.97, *t* = − 4.93, *p* < .001, 95% CI [–0.02, − 0.01]), and the composite index (B = − 0.06, *SE* = 0.01, β = –0.86, *t* = − 4.52, *p* < .001, 95% CI [–0.09, − 0.04]). On the other hand, the subjective-culture individualism-collectivism index (reversed) did not significantly predict COVID-19 cases per million (B = 0.00003, *SE* = 0.0002, β = 0.05, *t* = 0.15, *p* = .880, 95% CI [–0.0004, 0.0004]), COVID-19 deaths per million (B = − 0.00003, *SE* = 0.0004, β = –0.02, *t* = − 0.07, *p* = .941, 95% CI [–0.001, 0.001]), or the composite index (B = 0.0001, *SE* = 0.002, β = 0.02, *t* = 0.05, *p* = .959, 95% CI [–0.005, 0.005]).

**Fig. 1:**
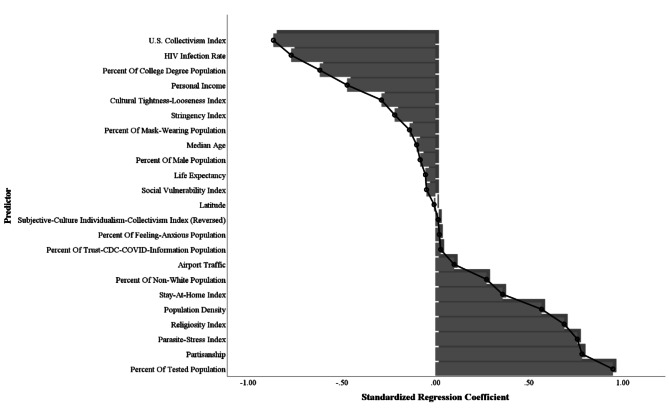
Standardized coefficients of variables predicting the composite COVID-19 severity index in a regression analysis in Study 1

To provide a visual representation of the effects of all independent variables and covariates on COVID-19 severity, Fig. 1 displays the standardized coefficient for each predictor of the composite COVID-19 severity index, encompassing dimensions of COVID-19 morbidity and mortality.

To assess the relationship between collectivism and COVID-19 severity while accounting for the shared variance explained by the covariates, residual analysis was conducted. Firstly, the confounding effects on COVID-19 cases, deaths, and the composite index were controlled by residualizing for all controlled variables. This process isolated the portion of COVID-19 severity not accounted for by the covariates. Subsequently, the ecological, socioeconomic-political factors, as well as religiosity and cultural tightness-looseness, were used to predict the two collectivism indices, isolating the unique variance in the collectivism indices not explained by these covariates.

**Fig. 2:**
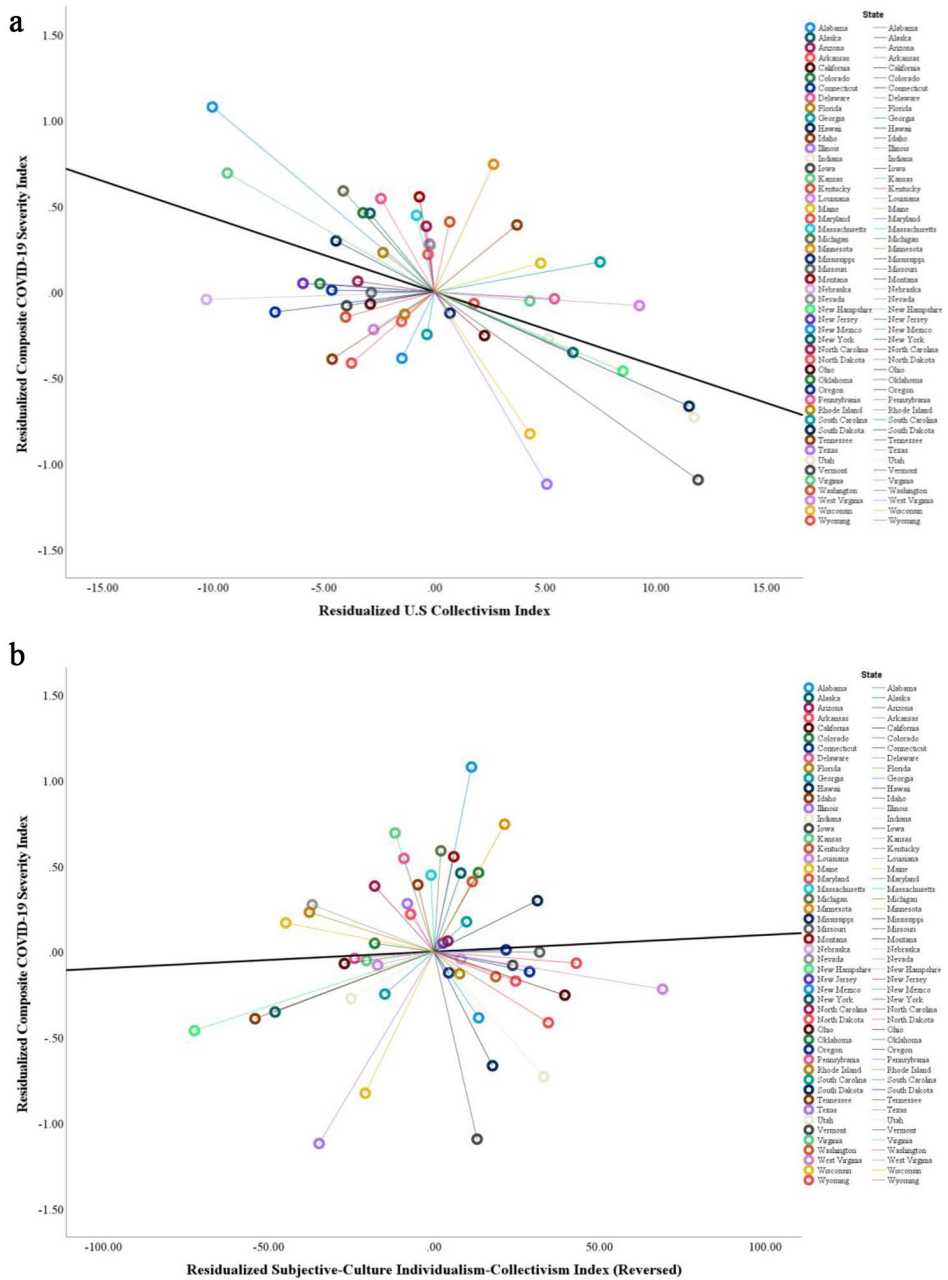
The scatterplots displaying the relationships between collectivism indices and composite COVID-19 severity index in residual analyses in Study 1. **a**. The scatterplot displaying the relationship between U.S. collectivism index and composite COVID-19 severity index in a residual analysis in Study 1 (*r* = –.52, *p* < .001, 95% CI [–0.69, –0.28]). **b**. The scatterplot displaying the relationship between subjective-culture individualism-collectivism index (reversed) and composite COVID-19 severity index in a residual analysis in Study 1 (*r* = .06, *p* = .678, 95% CI [–0.22, 0.33])

The residual analysis revealed that the residualized U.S collectivism index significantly and negatively predicted residualized COVID-19 cases per million (B = − 0.002, *SE* = 0.001, β = –0.34, *t* = − 2.41, *p* = .020, 95% CI [–0.0034, − 0.0003]), residualized COVID-19 deaths per million (B = − 0.01, *SE* = 0.002, β = –0.56, *t* = − 4.45, *p* < .001, 95% CI [–0.012, − 0.004]), and the residualized composite index (B = − 0.04, *SE* = 0.01, β = –0.53, *t* = − 4.18, *p* < .001, 95% CI [–0.07, − 0.02]). In contrast, the residualized subjective-culture individualism-collectivism index (reversed) did not significantly predict residualized COVID-19 cases per million (B = − 0.00001, *SE* = 0.0001, β = –0.01, *t* = − 0.10, *p* = .924, 95% CI [–0.0003, 0.0003]), residualized COVID-19 deaths per million (B = − 0.0002, *SE* = 0.0003, β = –0.08, *t* = − 0.60, *p* = .549, 95% CI [–0.0009, 0.0005]) and the residualized composite index (B = − 0.0001, *SE* = 0.0002, β = –0.05, *t* = − 0.43, *p* = .668, 95% CI [–0.005, 0.003]). Figure 2 provides a visual representation of the relationships between the collectivism indices and the composite index of COVID-19 severity.

Next, multilevel analysis was conducted to further investigate the OLS findings. The unconditional multilevel model, with the nine U.S. census divisions as a random intercept, revealed a non-significant likelihood-ratio test (LRT) of 2.56 × 10^–13^ (*p* = 1.00) and an intraclass correlation coefficient (ICC) of 0.000 for COVID-19 cases per million. However, for COVID-19 deaths per million, the LRT was significant at 9.77 (*p* = .002), with an ICC of 0.424. Similarly, the LRT for the composite COVID-19 severity index was significant at 4.04 (*p* = .044), with an ICC of 0.271. To maintain consistency in reporting statistical findings, multilevel analyses were performed for COVID-19 cases, deaths, and the composite index.

Table S2 presents the results of the multilevel models. In Model 1, before accounting for the effects of covariates, the U.S. collectivism index did not significantly predict COVID-19 cases per million (B = − 0.0001, *SE* = 0.001, *df* = 47, *t* = − 0.20, *p* = .844, 95% CI [–0.002, 0.001]), COVID-19 deaths per million (B = − 0.001, *SE* = 0.002, *df* = 47, *t* = − 1.07, *p* = .291, 95% CI [–0.005, 0.001]), or the composite index (B = − 0.01, *SE* = 0.01, *df* = 47, *t* = − 0.70, *p* = .490, 95% CI [–0.03, 0.01]). However, the subjective-culture individualism-collectivism index (reversed) significantly predicted COVID-19 cases per million (B = 0.0002, *SE* = 0.0001, *df* = 47, *t* = 2.75, *p* = .008, 95% CI [0.00006, 0.00040]), COVID-19 deaths per million (B = 0.001, *SE* = 0.0002, *df* = 47, *t* = 2.89, *p* = .006, 95% CI [0.0002, 0.0010]), and the composite index (B = 0.004, *SE* = 0.001, *df* = 47, *t* = 4.17, *p* < .001, 95% CI [0.002, 0.007]).

In Model 2, after accounting for the effects of covariates, the U.S collectivism index significantly predicted COVID-19 cases per million (B = − 0.003, *SE* = 0.001, *df* = 26, *t* = − 3.31, *p* = .003, 95% CI [–0.004, − 0.001]), COVID-19 deaths per million (B = − 0.01, *SE* = 0.002, *df* = 26, *t* = − 5.84, *p* < .001, 95% CI [–0.01, − 0.01]), and the composite index (B = − 0.06, *SE* = 0.01, *df* = 26, *t* = − 6.27, *p* < .001, 95% CI [–0.09, − 0.04]). However, the subjective-culture individualism-collectivism index (reversed) did not significantly predict COVID-19 cases per million (B = 0.00003, *SE* = 0.0001, *df* = 26, *t* = 0.21, *p* = .834, 95% CI [–0.0003, 0.0003]), COVID-19 deaths per million (B = 0.00003, *SE* = 0.0003, *df* = 26, *t* = 0.12, *p* = .903, 95% CI [–0.001, 0.001]), or the composite index (B = 0.0001, *SE* = 0.002, *df* = 26, *t* = 0.07, *p* = .943, 95% CI [–0.004, 0.004]).

Although the findings for the subjective-culture individualism-collectivism index (reversed) did not yield robust results, this study revealed a significant negative association between COVID-19 severity and the U.S. collectivism index, after controlling for various covariates. These findings suggested that the relationship between collectivism and COVID-19 severity in the United States could be influenced by other contextual variables. However, given the small sample size of Study 1, it was crucial to validate these findings with a larger sample, particularly when conducting a multilevel analysis. Therefore, the subsequent step involved examining how state-level collectivism would predict county-level COVID-19 severity while accounting for all contextual variables.

## Study 2

Study 2 aimed to bolster the findings of Study 1 by investigating the impact of state-level collectivism on county-level COVID-19 severity. This analysis accounted for a range of state- and county-level covariates. The research data and code for Study 2 can be accessed through the following OSF repository: https://osf.io/436zm/?view_only=5173a1bb043648afa76621d218e6f75c. Detailed information regarding the data sources and timeframes for the various variables can be found in Appendix 1 (refer to the Supplementary File).

### Method

#### Counties and timeframe

This study utilized data extracted from the New York Times dataset for a total of 3,133 counties that had identifiable FIPS codes and provided COVID-19 epidemiological information. The timeframe for data collection was identical to Study 1, ensuring consistency between the two studies.

#### Measures

##### County-level variables

###### COVID-19 severity indicators

The daily average of COVID-19 cases per million and COVID-19 deaths per million was calculated for each county. Similar to Study 1, the data were log-transformed to ensure appropriate statistical analysis and interpretation.

###### Covariates

The county-level covariates included in the analysis were as follows: stay-at-home index, latitude, parasites-stress index (derived from mortality rates of tuberculosis, diarrheal diseases, lower respiratory infections, meningitis, and hepatitis) [38], HIV infection rate, life expectancy, personal income, population density, percentage of male population, percentage of population with college degrees, social vulnerability index, partisanship, and rates of religious adherence per 1,000 population in 2010 (referred to as religiosity index).

##### State-level variables

In addition to the two collectivism indices examined in Study 1, the analysis in Study 2 included proxies of COVID-19-related prevention behaviors, percentage of non-white population, airport traffic, and the cultural tightness-looseness index from Study 1.

### Statistical approach

Given the nested structure of counties within states, a multilevel analysis was performed with states treated as a random intercept. The random-intercept multilevel model, aligned with the research objective, is represented by the following equations:

Level 1 (County-level):

Y_*ij*_ = B_0*j*_ + B_1_×Stay-at-home index_*ij*_ + B_2_×Latitude_*ij*_ + B_3_×Parasite-stress index_*ij*_ + B_4_×HIV infection rate_*ij*_ + B_5_×Life expectancy_*ij*_ + B_6_×Personal income_*ij*_ + B_7_×Population density_*ij*_ + B_8_×Percent male population_*ij*_ + B_9_×Percent college degree population_*ij*_ + B_10_×Median age_*ij*_ + B_11_×Social vulnerability index_*ij*_ + B_12_×Partisanship_*ij*_ + B_13_×Religiosity index_*ij*_ + e_*ij*_

Level 2 (State-level):

B_0*j*_ = γ_00_ + γ_01_×Stringency index_*j*_ + γ_02_×Percent tested for COVID_*j*_ + γ_03_×Percent wearing mask_*j*_ + γ_04_×Percent trust CDC information_*j*_ + γ_05_×Percent feeling anxious_*j*_ + γ_06_×Percent non-white population_*j*_ + γ_07_×Airport traffic_*j*_ + γ_08_×Cultural tightness-looseness index_*j*_ + γ_09_×Subjective-culture individualism-collectivism index (reversed)_*j*_ + γ_10_×U.S collectivism index_*j*_ + u_0*j*_.

In the equations above: Y_*ij*_ represents the COVID-19 severity in county *i* of state *j*. B_0*j*_ is the random intercept for state *j*, representing the average severity across counties within the state. B_1_ to B_13_ are the fixed-effect coefficients for the county-level predictors. γ_00_ represents the overall intercept for the state-level model. γ_01_ to γ_10_ are the fixed-effect coefficients for the state-level predictors. e_*ij*_ is the county-level residual term. u_0*j*_ is the state-level residual term.

### Results and discussion

The unconditional model demonstrated that state membership accounted for 43.5% (LRT = 934, *p* < .001), 48.9% (LRT = 1060, *p* < .001), and 55.9% (LRT = 1060, *p* < .001) of the variance in county-level COVID-19 cases per million, county-level COVID-19 deaths per million, and the county-level composite index of COVID-19 severity, respectively. These results supported the use of multilevel analyses [109, 110].

As shown in Table S3, in the absence of controlling for county- and state-level covariates (Model 1), subjective-culture individualism-collectivism index (reversed) exhibited a significant positive relationship with COVID-19 cases per million (B = 0.0004, *SE* = 0.0001, *df* = 3129, *t* = 3.84, *p* < .001, 95% CI [0.0002, 0.0006]), COVID-19 deaths per million (B = 0.001, *SE* = 0.0002, *df* = 3117, *t* = 6.25, *p* < .001, 95% CI [0.001, 0.001]), and the composite index (B = 0.005, *SE* = 0.001, *df* = 3129, *t* = 5.97, *p* < .001, 95% CI [0.003, 0.007]). On the other hand, U.S. collectivism index displayed a marginally negative association with COVID-19 cases per million (B = − 0.001, *SE* = 0.001, *df* = 3129, *t* = − 1.75, *p* = .080, 95% CI [–0.0031, 0.0002]), and COVID-19 deaths per million (B = − 0.003, *SE* = 0.002, *df* = 3117, *t* = − 1.78, *p* = .075, 95% CI [–0.0060, 0.0003]), but it significantly predicted the composite index of COVID-19 severity (B = − 0.02, *SE* = 0.01, *df* = 3129, *t* = − 2.91, *p* = .004, 95% CI [–0.04, − 0.01]).

**Fig. 3:**
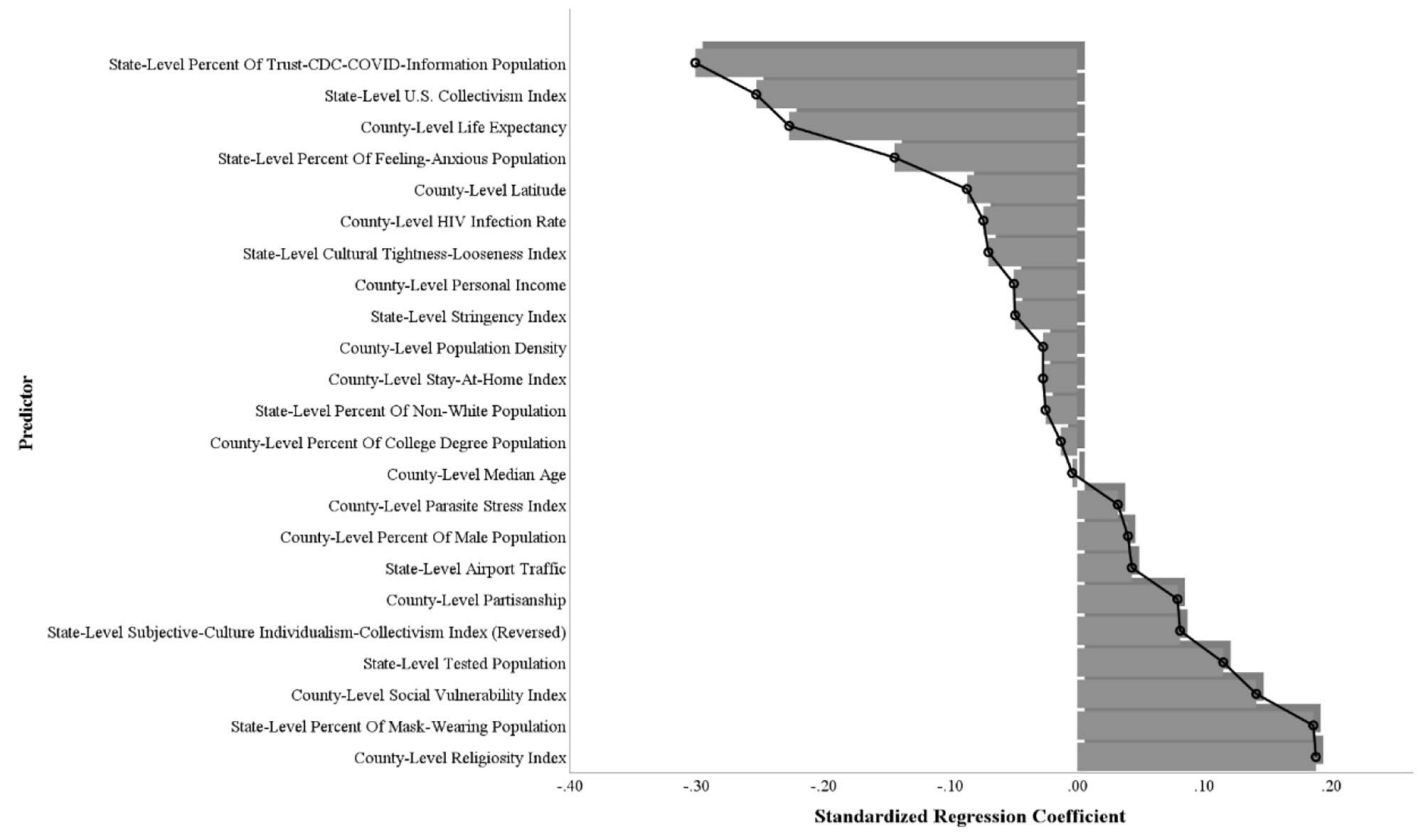
Standardized regression coefficients of predictors for the composite COVID-19 severity index in a multilevel analysis in Study 2

After accounting for the county- and state-level covariates, U.S. collectivism index demonstrated a significant negative relationship with COVID-19 cases per million (B = − 0.002, *SE* = 0.001, *df* = 2088, *t* = − 2.08, *p* = .037, 95% CI [–0.0045, − 0.0001]), COVID-19 deaths per million (B = − 0.01, *SE* = 0.002, *df* = 2088, *t* = − 2.59, *p* = .010, 95% CI [–0.010, − 0.001]), and the composite index (B = − 0.03, *SE* = 0.01, *df* = 2088, *t* = − 2.85, *p* = .004, 95% CI [–0.04, − 0.01]). However, there were non-significant associations found between subjective-culture individualism-collectivism index (reversed) and COVID-19 cases per million (B = 0.0003, *SE* = 0.002, *df* = 2088, *t* = 1.81, *p* = .070, 95% CI [–0.00002, 0.00064]), COVID-19 deaths per million (B = − 0.0002, *SE* = 0.0003, *df* = 2088, *t* = − 0.72, *p* = .470, 95% CI [–0.0009, 0.0004]), and the composite index (B = 0.001, *SE* = 0.001, *df* = 2088, *t* = 0.71, *p* = .476, 95% CI [–0.002, 0.004]). The standardized coefficients of county- and state-level predictors for the composite COVID-19 severity index are visualized in Fig. 3.

The findings from Study 2 reinforced the results of Study 1, providing further evidence that counties located in more collectivistic states demonstrated better control of COVID-19 when considering all relevant predictors of the pandemic. However, it was worth noting that neither Study 1 nor Study 2 examined the daily fluctuations in COVID-19 severity. Therefore, it was crucial to extend the analysis by nesting the daily COVID-19 epidemiological data within states to explore whether the impact of collectivism on COVID-19 severity would persist when accounting for the influences of daily-level predictors.

## Study 3

In Study 3, the focus was on investigating the impact of state-level collectivism on daily-level COVID-19 severity, while controlling for various daily- and state-level covariates. To accomplish this, the study considered time trends, seasonality, and serial autocorrelation of the daily COVID-19 data, as well as considered the effects of state-level covariates associated with COVID-19 severity. The research data and code can be accessed through the following OSF repository: https://osf.io/436zm/?view_only=5173a1bb043648afa76621d218e6f75c. Details about the data sources can be found in Appendix 1 (see Supplementary File).

### Method

#### States and time series data

Daily epidemiological data were obtained from the New York Times dataset. Due to variations in the initial reporting dates of COVID-19 epidemiological data, the time frames for each state differed, resulting in a total of 52,806 observations (i.e., days) for the multilevel dataset.

#### Measures

##### Daily-level variables

In Study 3, the log-transformed daily COVID-19 cases per million and deaths per million were computed. To capture the dynamics of COVID-19 severity over time, the previous day’s (day *x* – 1) epidemiological data were used to predict the data for the current day (day *x*) [38, 111, 112]. The influence of time trends on COVID-19 severity was accounted for by including the day as a covariate. Additionally, the impact of seasonality, which has been linked to COVID-19 severity [113–115], was addressed by coding autumn and winter days as “1” and other days as “0”. The daily-level analysis also incorporated the stay-at-home index and stringency index.

##### State-level variables

The state-level variables used in Study 3 were the same as those utilized in Study 1.

### Statistical approach

This study adopted the analytical approach of recent studies by nesting the time series data within states [38, 51, 105]. To address the research aim, a random-intercept multilevel model was employed, described by the following equations:

Level 1 (Daily-level):

Y_*ij*_ = B_0*j*_ + B_1_×Preceding-day COVID-19 severity_*ij*_ + B_2_×Day_*ij*_ + B_3_×Season_*ij*_ + B_4_×Stay-at-home index_*ij*_ + B_5_×Stringency index_*ij*_ + e_*ij*_

Level 2 (State-level):

B_0*j*_ = γ_00_ + γ_01_×Percent tested for COVID_*j*_ + γ_02_×Percent wearing mask_*j*_ + γ_03_×Percent trust CDC information_*j*_ + γ_04_×Percent feeling anxious_*j*_ + γ_05_×Latitude_*j*_ + γ_06_×Parasite-stress index_*j*_ + γ_07_×HIV infection rate_*j*_ + γ_08_×Personal income_*j*_ + γ_09_×Population density_*j*_ + γ_10_×Median age_*j*_ + γ_11_×Percent male population_*j*_ + γ_12_×Percent college degree population_*j*_ + γ_13_×Life expectancy_*j*_ + γ_14_×Social vulnerability index_*j*_ + γ_15_×Airport traffic_*j*_ + γ_16_×Percent non-white population_*j*_ + γ_17_×Partisanship_*j*_ + γ_18_×Religiosity index_*j*_ + γ_19_×Cultural tightness-looseness index_*j*_ + γ_20_×Subjective-culture individualism-collectivism index (reversed)_*j*_ + γ_21_×U.S. collectivism index_*j*_ + u_0*j.*_

In the equations above: Y_*ij*_ represents COVID-19 severity in day *i* of state *j*. B_0*j*_ is the random intercept for state *j*, representing the average severity across days within the state. B_1_ to B_5_ are the fixed-effect coefficients for the daily-level predictors. γ_00_ represents the overall intercept for the state-level model. γ_01_ to γ_21_ are the fixed-effect coefficients for the state-level predictors. e_*ij*_ is the daily-level residual term. u_0*j*_ is the state-level residual term.

### Results and discussion

The unconditional models revealed that a significant portion of the total variance in COVID-19 cases per million (3.23%, LRT = 1094, *p* < .001), COVID-19 deaths per million (8.48%, LRT = 2184, *p* < .001), and the composite COVID-19 severity index (4.54%, LRT = 1646, *p* < .001) could be attributed to state-level differences. This provided a rationale for conducting multilevel analysis [116, 117].

Before accounting for daily- and state-level covariates, Table S4 reveals that the U.S. collectivism index had a non-significant negative effect on COVID-19 cases per million (B = − 0.001, *SE* = 0.001, *df* = 40,414, *t* = − 0.61, *p* = .544, 95% CI [–0.004, 0.002]), COVID-19 deaths per million (B = − 0.003, *SE* = 0.002, *df* = 32,868, *t* = − 1.92, *p* = .054, 95% CI [–0.00705, 0.00006]), and the composite COVID-19 severity index (B = − 0.002, *SE* = 0.003, *df* = 40,675, *t* = − 0.87, *p* = .386, 95% CI [–0.007, 0.003]). Conversely, the subjective-culture individualism-collectivism index (reversed) significantly predicted COVID-19 cases per million (B = 0.0004, *SE* = 0.0002, *df* = 40,414, *t* = 2.27, *p* = .023, 95% CI [0.00005, 0.00070]), COVID-19 deaths per million (B = 0.001, *SE* = 0.0002, *df* = 32,868, *t* = 2.54, *p* = .011, 95% CI [0.0001, 0.0009]), and the composite index (B = 0.001, *SE* = 0.0003, *df* = 40,675, *t* = 2.74, *p* = .006, 95% CI [0.0002, 0.0013]).

**Fig. 4:**
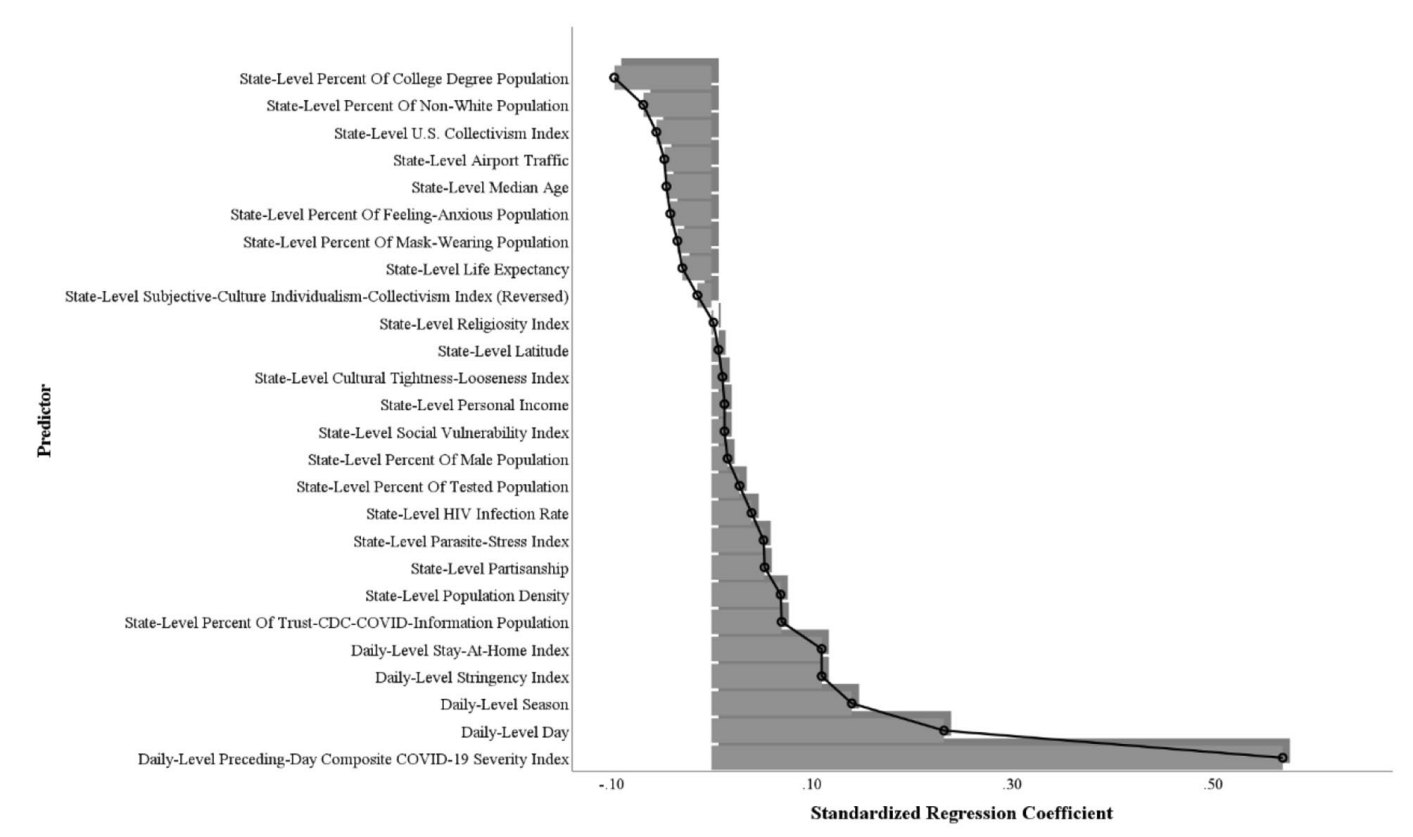
Standardized coefficients of predictors for the composite COVID-19 severity index in a multilevel analysis in Study 3

However, when considering the effects of daily- and state-level covariates, the U.S. collectivism index exhibited a significant negative association with COVID-19 cases per million (B = − 0.002, *SE* = 0.001, *df* = 35,198, *t* = − 3.10, *p* = .002, 95% CI [–0.004, − 0.001]), COVID-19 deaths per million (B = − 0.003, *SE* = 0.001, *df* = 25,767, *t* = − 2.35, *p* = .019, 95% CI [–0.0053, − 0.0005]), and the composite index (B = − 0.005, *SE* = 0.002, *df* = 35,458, *t* = − 3.18, *p* = .001, 95% CI [–0.008, − 0.002]). On the other hand, the subjective-culture individualism-collectivism index (reversed) did not significantly predict COVID-19 cases per million (B = − 0.000003, *SE* = 0.0001, *df* = 35,198, *t* = − 0.02, *p* = .980, 95% CI [–0.0003, 0.0003]), COVID-19 deaths per million (B = − 0.0002, *SE* = 0.0002, *df* = 25,765, *t* = − 0.78, *p* = .433, 95% CI [–0.0006, 0.0003]), or the composite index (B = − 0.0001, *SE* = 0.0003, *df* = 35,458, *t* = − 0.50, *p* = .620, 95% CI [–0.0007, 0.0004]). Figure 4 illustrates the standardized coefficients of all predictors in the multilevel analysis.

Given the compelling evidence regarding the significant impact of the U.S. collectivism index on COVID-19 severity across multiple studies, conducting a comprehensive meta-analysis became imperative to further explore and validate these findings. The purpose of this meta-analysis was to systematically analyze and synthesize the results from various studies.

## Study 4

Study 4 conducted a mini meta-analysis to enhance the credibility and validity of the observed relationships between collectivism and COVID-19, thereby providing a more robust evaluation of their collective impact and generalizability across Studies 1 to 3. By undertaking this comprehensive examination, the study aimed to deepen our understanding the relationship between collectivism and COVID-19 pandemic in the United States.

**Fig. 5:**
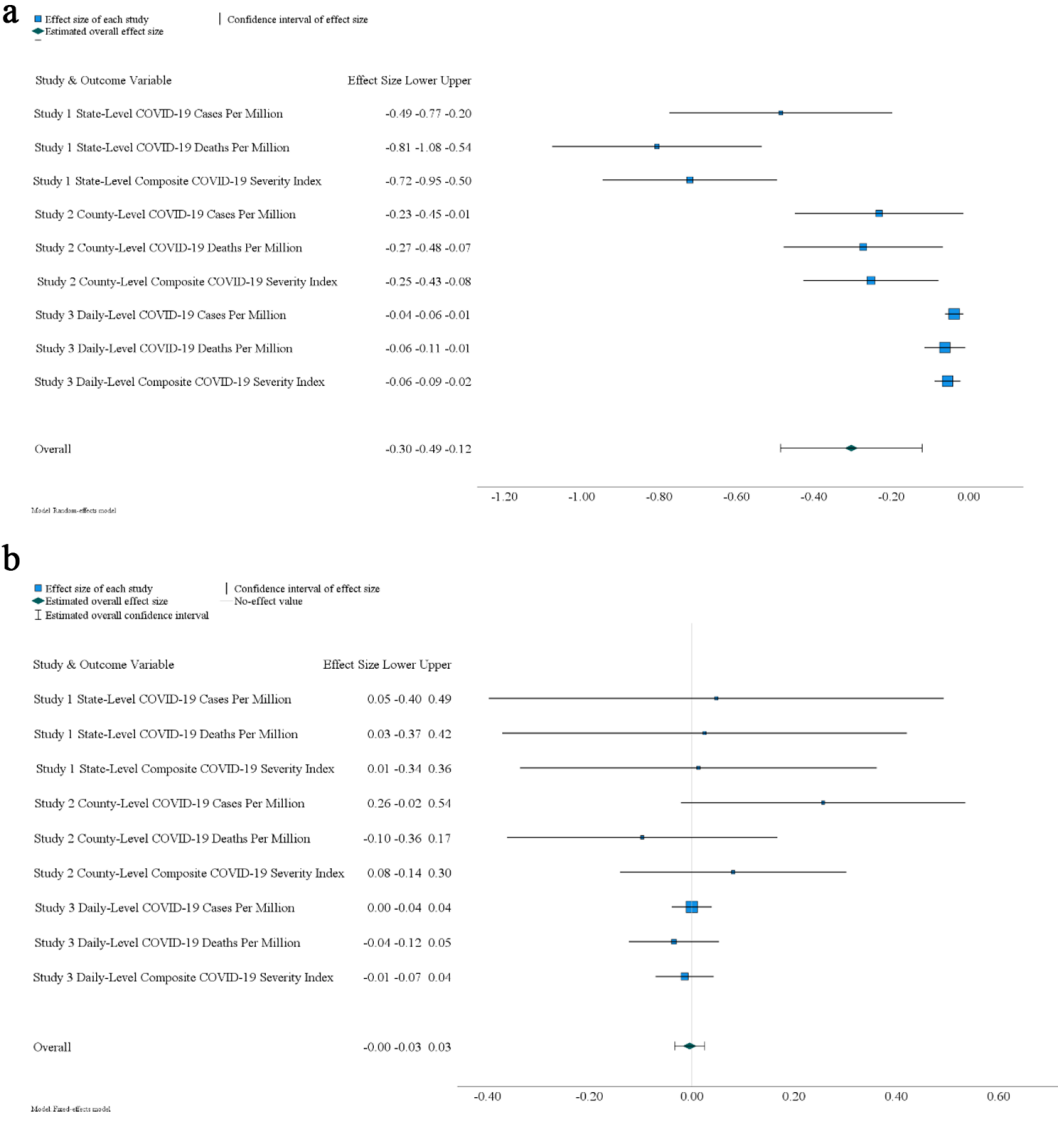
Forest plots in Study 4. Note. Standardized regression coefficients are reported. **a**. The distribution of standardized regression coefficients of U.S collectivism index predicting COVID-19 severity in multilevel analyses across different studies. **b.** The distribution of standardized regression coefficients of subjective-culture individualism-collectivism index (reversed) predicting COVID-19 severity in multilevel analyses across different studies

The first mini meta-analysis aimed to determine the significance of the overall effect of the U.S collectivism index on COVID-19 severity across studies, considering covariates in multilevel analyses across Studies 1 to 3. Each study (i.e., Study 1, Study 2, and Study 3) assessed COVID-19 severity using three outcome variables: COVID-19 cases per million, COVID-19 deaths per million, and the composite index of COVID-19 severity. Meta-analysis was conducted to combine the standardized regression coefficients of the U.S collectivism index across the three studies. Fig. 5a depicts significant heterogeneity in the effects across the studies (*Q*(8) = 85.33, *p* < .001), indicating the need for a random-effect meta-analysis with a restricted maximum likelihood method. The results demonstrated a significant combined effect, indicating that the U.S collectivism index was associated with a mean effect size of –0.30 (*SE* = 0.09, *z* = − 3.25, *p* = .001, 95% CI [–0.49, –0.12]).

The second mini meta-analysis aimed to assess the significance of the overall effect of the subjective-culture individualism-collectivism index (reversed) on COVID-19 severity across studies, while considering covariates in each study. Fig. 5b demonstrates non-significant heterogeneity in effects across studies (*Q*(8) = 5.13, *p* = .743). Consequently, a fixed-effect model was utilized, revealing a negative but non-significant combined effect across studies: *Overall Effect* = –0.004, *SE* = 0.01, *z* = − 0.27, *p* = .785, 95% CI [–0.03, 0.03], suggesting that any observed differences in effect sizes between the studies were likely due to chance (see Fig. 5b).

The findings provided support for the significant impact of state-level collectivism, as indicated by the U.S. collectivism index, on COVID-19 severity across various levels including state, county, and daily analyses. Additionally, the results indicated that the subjective-culture individualism-collectivism index (reversed) might not exert a substantial influence on COVID-19 severity, considering the available evidence from the included studies and the covariates considered in the analyses.

## General discussion

This research, consisting of four studies, indicates that state-level collectivism, as measured by the objective cultural collectivism index [101], was a significant and negative predictor of COVID-19 severity at the state, county, and daily levels after accounting for important confounders. However, the subjective cultural collectivism index [102] showed no significant relationship with COVID-19 severity across the studies, once the confounding variables were taken into account. These findings align with the parasite-stress theory of sociality, suggesting that collectivism acts as a trait of ingroup assortative sociality, offering defense against infectious diseases [10, 39]. Thus, the results of this study support the proactive role of collectivism in defending against the novel coronavirus, consistent with recent group-level studies [31–35, 40, 41].

From a theoretical perspective, the current findings that collectivism negatively predicted COVID-19 severity after accounting for covariates are consistent with the principles of the parasite-stress theory of sociality. According to this theory, collectivism is seen as a characteristic of ingroup assortative sociality, which plays a crucial role in defending against infectious diseases [10, 11, 118, 119]. The parasite-stress theory suggests that in environments with a higher prevalence of infectious diseases, individuals and groups are more likely to adopt behavioral and social strategies aimed at reducing the risk of infection and maintaining overall health. Collectivism, as an adaptive response to heightened disease threats, encourages cooperation, interdependence, and adherence to group norms and practices that help to mitigate the transmission and impact of infectious diseases [45].

However, it is essential to emphasize that adjusting for confounding variables is crucial to identify the true negative association between collectivism, as indicated by an objective collectivism index, and COVID-19 severity. While recent studies have explored the relationship between collectivism and the COVID-19 pandemic at a group level using observational data [31–36, 38], it is imperative to account for confounding variables to establish a robust relationship [54, 55].

Although some significant covariates such as GDP per capita, population density, and percentage of non-white population have been controlled for in recent state-level analyses [36], it is still possible for important confounding effects to mask the true relationship. For instance, collectivist cultures often prioritize social cohesion and group well-being, which can lead to higher compliance with preventive measures [45, 47–49]. Therefore, the present study has adjusted for the effects of protective behaviors to reveal a clearer and more direct relationship between collectivism and COVID-19 severity.

Furthermore, the inclusion of additional variables such as the historical parasite-stress index, HIV infection rate, latitude, and social vulnerability index enables the study to account for previous exposure to infectious diseases, environmental conditions, social vulnerability [57, 70, 71], and other theoretical explanations for the observed relationship, such as the life history strategy theory [72–74]. Moreover, considering that cultural tightness-looseness and religiosity are closely associated with the concept of collectivism [13, 37, 45, 102], adjusting for these cultural factors allows for isolating the unique association between collectivism and COVID-19 severity.

Therefore, this analysis underscores the importance of considering a comprehensive range of covariates when examining the relationship between collectivism and COVID-19 severity. By accounting for relevant factors, researchers can gain a more nuanced understanding of the complex interplay between cultural collectivism and its impact on COVID-19 severity.

To gain a more comprehensive understanding of the negative impact of the U.S. collectivism index on COVID-19 severity, this research examines the objective indicators utilized in constructing this index, considering confounding variables. One such indicator is living arrangement, which can be influenced by socioeconomic development and disease protective behaviors. Socioeconomic factors play a significant role in determining living arrangements [120, 121], as individuals with higher socioeconomic status are more likely to live alone. Furthermore, disease protective behaviors, such as adherence to public health guidelines and engagement in preventive measures, might also impact the proportion of individuals living alone. For instance, regions with a higher percentage of individuals who prioritize disease prevention may exhibit a greater proportion of people living alone, as they might prioritize social distancing and minimizing close contact as precautionary measures.

Therefore, it is crucial to consider that a region with a lower proportion of people living alone may exhibit lower COVID-19 severity due to factors such as better access to healthcare, higher socioeconomic status, or greater adherence to disease protective behaviors. By accounting for these confounding factors, the present research provides additional insights into the negative association between the U.S. collectivism index and COVID-19 severity. This underscores the significance of considering socioeconomic factors and disease protective behaviors in order to disentangle the unique contributions of collectivism and other factors in explaining variations in COVID-19 outcomes.

Moreover, the marriage to divorce ratio, serving as an objective indicator of the U.S. collectivism index, offers insights into the societal stability of family structures and social cohesion [77, 101]. However, it is crucial to consider and address confounding factors that may influence both the marriage to divorce ratio and COVID-19 severity. One such confounder is cultural tightness-looseness, which independently impacts the ratio due to variations in attitudes towards marriage and divorce [122, 123]. Additionally, cultural tightness-looseness has been identified as a strong predictor of COVID-19 outcomes [52, 124].

Furthermore, socioeconomic development acts as another confounding factor, as higher levels of development can contribute to more stable family structures through increased economic security and access to resources [125, 126]. Moreover, political factors, including government policies and legal frameworks [127, 128], can also influence the ratio. By meticulously controlling for cultural norms, socioeconomic and political factors, this research isolates the specific effect of the marriage to divorce ratio on COVID-19 severity in relation to collectivism.

Another noteworthy objective indicator is the percentage of people carpooling to work, which provides insights into commuting patterns. The availability and quality of transportation infrastructure have been identified as influential factors in determining these patterns [129, 130]. Regions with well-developed public transportation systems generally exhibit lower rates of carpooling, irrespective of collectivism. Conversely, areas with limited transportation options tend to have higher rates of carpooling.

Furthermore, higher levels of socioeconomic development are associated with a greater proportion of individuals owning private vehicles and choosing individual commuting, which reduces the likelihood of carpooling [131]. Thus, by controlling for these factors in the present research, the specific effect of carpooling influenced by collectivism on COVID-19 severity is isolated.

The percentage of voters supporting the Libertarian Party (reversed) serves as another indicator of the U.S. collectivism index. By adjusting for the effects of political factors in the current research, we aim to uncover the specific impact of collectivism on COVID-19 severity, beyond the influence of politics.

Political ideologies can significantly shape individual behaviors, including adherence to disease preventive measures [65–68]. Collectivist political ideologies, for example, may prioritize community well-being and foster greater compliance with public health guidelines. On the other hand, individualistic political ideologies may place a stronger emphasis on personal freedom and autonomy, potentially influencing behaviors that can impact COVID-19 severity.

Through the control of political factors, this research isolates the specific impact of collectivism on COVID-19 severity, providing a clearer understanding of the relationship between collectivism as a cultural construct and COVID-19 severity, independent of political influences.

The percentage of self-employed workers (reversed) serves as a valuable indicator of economic factors and individual autonomy within a society. However, it is crucial to consider the influence of socioeconomic development on the employment landscape, as higher levels of development can lead to a reduction in self-employed workers [132], regardless of collectivism. Additionally, the level of college education can act as a confounding factor in the analysis of self-employment [133].

Moreover, industry-specific variables, gender, and age groups can contribute to variations in the prevalence of self-employment [134]. By controlling for a series of socioeconomic development factors, the present research isolates the specific impact of self-employment influenced by collectivism on COVID-19 severity, providing a deeper understanding of the relationship between these variables.

When examining the relationship between subjective-culture individualism-collectivism index (reversed) and COVID-19 severity while considering the effects of confounding variables, the results have been intriguing. Across multiple studies, the findings indicate that subjective cultural collectivism, as measured by the subjective-culture individualism-collectivism index (reversed), did not demonstrate a significant relationship with COVID-19 severity. This implies that the specific beliefs and values encompassed by subjective cultural collectivism (religiosity, opposition to abortion, and opposition to same-sex marriage) [102] may not directly influence the outcomes of the pandemic. This raises thought-provoking questions about the role of subjective cultural collectivism in shaping COVID-19 outcomes.

The absence of a significant relationship between subjective cultural collectivism and COVID-19 severity after adjusting for confounding effects prompts us to explore alternative factors that may be more closely associated with the spread and impact of the virus. While subjective cultural collectivism captures important aspects of a society’s collective mindset, such as religious devotion and social attitudes, it appears that other factors play a more prominent role in determining the severity of the pandemic.

One possible explanation for the lack of significance in the relationship could be attributed to the strong correlation observed between the subjective cultural collectivism index and the confounding variables controlled for in the current research. The study conducted by M Minkov and A Kaasa [102] provides valuable insights into the associations between their individualism-collectivism index and various socio-political-cultural factors, shedding light on the complexities underlying the relationship.

The positive correlations between the subjective individualism-collectivism index and median household income (*r* = .67) and GDP per capita (*r* = .54) suggest that higher economic status may be linked to higher levels of subjective cultural individualism. This implies that individuals with greater financial resources are more likely to adopt cultural values and beliefs that emphasize personal autonomy and independence. It is plausible that individuals who are economically well-off have the means to pursue individualistic aspirations and prioritize their own needs and desires.

On the other hand, the negative correlations between the subjective cultural collectivism index and the percentage of votes for Trump in both the 2016 (*r* = − .77) and 2020 (*r* = − .79) elections suggest that political ideologies and affiliations can influence the subjective aspect of cultural individualism. This implies that individuals with different political leanings may prioritize distinct cultural values and expressions of individualism. Political ideologies often shape individuals’ attitudes and behaviors, including their propensity to embrace personal freedom and autonomy. Therefore, the political landscape can be a significant factor in understanding the relationship between subjective cultural individualism and various outcomes.

Moreover, the strong negative correlation between the subjective-culture individualism-collectivism index and the cultural tightness-looseness index (*r* = − .87) highlights the interplay between cultural norms and the subjective perception of individualism. Culturally tight societies, characterized by strict norms and practices, may discourage individualistic attitudes and behaviors, resulting in lower levels of subjective cultural individualism. Conversely, culturally loose societies may foster an environment that values personal freedom and diverse expressions of cultural values, leading to higher levels of subjective cultural individualism. These findings suggest that the degree of cultural tightness or looseness can shape individuals’ subjective experiences of individualism and their behaviors related to various outcomes.

Considering these correlations, it becomes evident that the subjective cultural collectivism index is closely intertwined with various socio-political-cultural factors. The high correlations observed between this index and confounding variables indicate that the influence of subjective cultural collectivism on COVID-19 pandemic might be mediated or confounded by these related factors. However, it is worth noting that the subjective cultural collectivism index differs from the U.S. collectivism index in terms of the indicators used and the nature of the construct it captures. While the U.S. collectivism index focuses on objective indicators related to social interconnectedness and interdependence, the subjective-culture individualism-collectivism index (reversed) captures a more subjective aspect of cultural collectivism, reflecting attitudes and beliefs related to religion, abortion, and same-sex marriage. Hence, despite its non-significance in relation to COVID-19 severity in the current research, the subjective cultural collectivism index can provide complementary insights into the broader understanding of cultural collectivism and its potential influence on various outcomes.

Several limitations should be acknowledged and addressed in future studies. The use of area-level analyses in this study raises concerns about the ecological fallacy issue [135] when interpreting the results in relation to previous research [31–36, 40, 41]. To overcome this limitation, future studies should investigate collectivism at the individual level to examine potential differences between COVID-19 infected and non-infected individuals. Such investigations would allow testing the hypothesis that high levels of collectivism are effective in avoiding and managing infectious diseases [10].

Furthermore, the critical variables such as individual educational attainment, age, gender, family status, and other influential factors identified by survey researchers were excluded from the current aggregate group-level analyses. Therefore, while the aggregate data analyses offer valuable insights at the population level, it is essential to acknowledge the limitations stemming from the exclusion of individual-level variables. The impact of omitted variables should be considered when interpreting the findings. Moreover, it is emphasized that the absence of individual-level variables in the current analyses does not diminish their importance. On the contrary, it underscores the need for future research to incorporate individual-level data to further understand the influence of variables such as educational attainment, age, gender, family status, and other relevant factors identified in previous studies. This would facilitate a more comprehensive understanding of the multifaceted factors that underlie public health behaviors and decision-making processes.

Additionally, previous research [50] utilized micro-level self-report data on ancestors’ country of origin to infer intergenerational transmitted cultural traits of Hofstede’s cultural individualism [136] as a measure of county-level collectivism. Replicating the present research using this approach would provide an opportunity to cross-validate the current findings.

## Conclusion

This research contributes empirical evidence that supports the inverse relationship between state-level collectivism, as measured by the U.S. collectivism index, and the severity of COVID-19 after controlling for crucial confounding factors. These findings are consistent with the parasite-stress theory of sociality, which posits that collectivism serves as a characteristic of ingroup assortative sociality, providing a defense mechanism against infectious diseases. However, it should be noted that the subjective-cultural individualism-collectivism index (reversed) did not exhibit a statistically significant association with COVID-19 severity when accounting for confounding variables, potentially due to the high correlations between the index and the controlled variables in the analysis.

### Electronic supplementary material

Below is the link to the electronic supplementary material.

Supplementary Material 1

## Data Availability

The datasets generated and/or analysed during the current study are available in the Open Science Framework repository, https://osf.io/436zm/?view_only=5173a1bb043648afa76621d218e6f75c.
