# Supplementary material for: Beyond the surface: accounting for confounders in understanding the link between collectivism and COVID-19 pandemic in the United States
Source: BMC Public Health. 2023 Aug 9;23:1513. doi: 10.1186/s12889-023-16384-2 (PMC10413761; doi:10.1186/s12889-023-16384-2)
Supplement: Supplementary file 1 — Supplementary Material 1 [file 12889_2023_16384_MOESM1_ESM.docx]

Table S1. OLS regression analysis results in Study 1

| Predictor | Outcome variable | | | | | | | | | | | | | | | | | | | | | | | | | | | | |
| --- | --- | --- | --- | --- | --- | --- | --- | --- | --- | --- | --- | --- | --- | --- | --- | --- | --- | --- | --- | --- | --- | --- | --- | --- | --- | --- | --- | --- | --- |
|  | COVID–19 cases per million | | | | | | | | |  | COVID–19 deaths per million | | | | | | | | |  | Composite COVID–19 severity index | | | | | | | | |
|  | Model 1 without covariates | | | |  | Model 2 with covariates | | | |  | Model 1 without covariates | | | |  | Model 2 with covariates | | | |  | Model 1 without covariates | | | |  | Model 2 with covariates | | | |
|  | B | *SE* | β | *t* |  | B | *SE* | β | *t* |  | B | *SE* | β | *t* |  | B | *SE* | β | *t* |  | B | *SE* | β | *t* |  | B | *SE* | β | *t* |
| Constant | 2.49 | 0.04 |  | 60.04^***^ |  | 4.78 | 1.78 |  | 2.69^*^ |  | 0.56 | 0.08 |  | 7.09^***^ |  | 6.24 | 3.74 |  | 1.67 |  | 0.35 | 0.49 |  | 0.71 |  | 39.63 | 22.47 |  | 1.76 |
| Subjective-cultural individualism-collectivism index (reversed) | 0.0002 | 0.0001 | .37 | 2.67^*^ |  | 0.00003 | 0.0002 | .05 | 0.15 |  | 0.001 | 0.0002 | .51 | 3.96^***^ |  | –0.00003 | 0.0004 | –.02 | –0.07 |  | 0.004 | 0.001 | .52 | 4.05 |  | 0.0001 | 0.002 | .02 | 0.05 |
| U.S. collectivism index | –0.0001 | 0.001 | –.03 | –0.19 |  | –0.003 | 0.001 | –.49 | –2.39^*^ |  | –0.001 | 0.002 | –.12 | –0.93 |  | –0.01 | 0.002 | –.97 | –4.93^***^ |  | –0.01 | 0.01 | –.09 | –0.67 |  | –0.06 | 0.01 | –.86 | –4.52^***^ |
| Stay–at–home index |  |  |  |  |  | 0.002 | 0.001 | .40 | 1.33 |  |  |  |  |  |  | 0.002 | 0.003 | .21 | 0.72 |  |  |  |  |  |  | 0.02 | 0.02 | .36 | 1.29 |
| Stringency index |  |  |  |  |  | –0.002 | 0.003 | –.10 | –0.67 |  |  |  |  |  |  | –0.01 | 0.01 | –.26 | –1.79 |  |  |  |  |  |  | –0.05 | 0.03 | –.22 | –1.52 |
| %COVID–19 tested |  |  |  |  |  | 0.03 | 0.004 | 1.31 | 7.39^***^ |  |  |  |  |  |  | 0.01 | 0.01 | .29 | 1.74 |  |  |  |  |  |  | 0.29 | 0.05 | .95 | 5.78^***^ |
| %Mask wearing |  |  |  |  |  | 0.000001 | 0.003 | .0001 | 0.0004 |  |  |  |  |  |  | –0.01 | 0.01 | –.24 | –0.83 |  |  |  |  |  |  | –0.02 | 0.04 | –.14 | –0.51 |
| %Trust the CDC information |  |  |  |  |  | 0.001 | 0.01 | .11 | 0.26 |  |  |  |  |  |  | –0.002 | 0.01 | –.06 | –0.15 |  |  |  |  |  |  | 0.005 | 0.06 | .03 | 0.07 |
| %Feeling anxious |  |  |  |  |  | –0.004 | 0.01 | –.10 | –0.46 |  |  |  |  |  |  | 0.01 | 0.02 | .14 | 0.65 |  |  |  |  |  |  | 0.01 | 0.12 | .02 | 0.10 |
| Latitude |  |  |  |  |  | 0.0003 | 0.001 | .07 | 0.65 |  |  |  |  |  |  | –0.001 | 0.001 | –.09 | –0.80 |  |  |  |  |  |  | –0.0005 | 0.01 | –.01 | –0.07 |
| Parasite–stress index |  |  |  |  |  | 0.004 | 0.003 | .42 | 1.65 |  |  |  |  |  |  | 0.02 | 0.01 | .87 | 3.60^**^ |  |  |  |  |  |  | 0.11 | 0.03 | .76 | 3.24^**^ |
| HIV infection rate |  |  |  |  |  | –0.01 | 0.004 | –.65 | –2.05 |  |  |  |  |  |  | –0.02 | 0.01 | –.65 | –2.15^*^ |  |  |  |  |  |  | –0.14 | 0.05 | –.77 | –2.62^*^ |
| Life expectancy |  |  |  |  |  | –0.002 | 0.01 | –.05 | –0.15 |  |  |  |  |  |  | –0.002 | 0.02 | –.04 | –0.10 |  |  |  |  |  |  | –0.02 | 0.14 | –.05 | –0.16 |
| Personal income |  |  |  |  |  | –0.54 | 0.26 | –.59 | –2.12^*^ |  |  |  |  |  |  | –0.42 | 0.54 | –.21 | –0.77 |  |  |  |  |  |  | –5.93 | 3.25 | –.47 | –1.82 |
| Population density |  |  |  |  |  | 0.04 | 0.03 | .37 | 1.49 |  |  |  |  |  |  | 0.13 | 0.05 | .59 | 2.50^*^ |  |  |  |  |  |  | 0.78 | 0.32 | .57 | 2.48^*^ |
| Median age |  |  |  |  |  | –0.01 | 0.01 | –.19 | –0.95 |  |  |  |  |  |  | 0.001 | 0.01 | .02 | 0.11 |  |  |  |  |  |  | –0.04 | 0.07 | –.10 | –0.54 |
| %Male population |  |  |  |  |  | 1.07 | 2.21 | .13 | 0.48 |  |  |  |  |  |  | –4.78 | 4.64 | –.27 | –1.03 |  |  |  |  |  |  | –8.88 | 27.93 | –.08 | –0.32 |
| %College degree population |  |  |  |  |  | –0.50 | 0.37 | –.43 | –1.36 |  |  |  |  |  |  | –1.61 | 0.78 | –.62 | –2.06^*^ |  |  |  |  |  |  | –9.95 | 4.68 | –.62 | –2.13^*^ |
| Social vulnerability index |  |  |  |  |  | –0.03 | 0.09 | –.07 | –0.35 |  |  |  |  |  |  | –0.01 | 0.19 | –.01 | –0.03 |  |  |  |  |  |  | –0.28 | 1.17 | –.05 | –0.24 |
| Airport traffic |  |  |  |  |  | 0.65 | 0.79 | .14 | 0.82 |  |  |  |  |  |  | 0.29 | 1.66 | .03 | 0.17 |  |  |  |  |  |  | 6.30 | 9.98 | .10 | 0.63 |
| %Non–white population |  |  |  |  |  | 0.003 | 0.12 | .01 | 0.02 |  |  |  |  |  |  | 0.39 | 0.26 | .46 | 1.52 |  |  |  |  |  |  | 1.44 | 1.54 | .27 | 0.94 |
| Partisanship |  |  |  |  |  | 0.36 | 0.12 | .85 | 2.92^**^ |  |  |  |  |  |  | 0.45 | 0.26 | .48 | 1.75 |  |  |  |  |  |  | 4.57 | 1.56 | .78 | 2.93^**^ |
| Religiosity index |  |  |  |  |  | 0.002 | 0.001 | .67 | 2.14^*^ |  |  |  |  |  |  | 0.004 | 0.002 | .50 | 1.68 |  |  |  |  |  |  | 0.03 | 0.01 | .69 | 2.38^*^ |
| Cultural tightness–looseness index |  |  |  |  |  | –0.001 | 0.001 | –.16 | –0.73 |  |  |  |  |  |  | –0.004 | 0.002 | –.33 | –1.55 |  |  |  |  |  |  | –0.02 | 0.01 | –.29 | –1.41 |

*Note*. ^*^*p* < .05, ^**^*p* < .01, ^***^*p* < .001.

Table S2. Multilevel analysis results in Study 1

| Predictor | Outcome variable | | | | | | | | | | | | | | | | | | | | | | | | | | | | |
| --- | --- | --- | --- | --- | --- | --- | --- | --- | --- | --- | --- | --- | --- | --- | --- | --- | --- | --- | --- | --- | --- | --- | --- | --- | --- | --- | --- | --- | --- |
|  | COVID–19 cases per million | | | | | | | | |  | COVID–19 deaths per million | | | | | | | | |  | Composite COVID–19 severity index | | | | | | | | |
|  | Model 1 without covariates  ICC = .000 | | | |  | Model 2 with covariates  ICC = .000 | | | |  | Model 1 without covariates  ICC = .276 | | | |  | Model 2 with covariates  ICC = .398 | | | |  | Model 1 without covariates  ICC = .000 | | | |  | Model 2 with covariates  ICC = .114 | | | |
|  | B | *SE* | *df* | *t* |  | B | *SE* | *df* | *t* |  | B | *SE* | *df* | *t* |  | B | *SE* | *df* | *t* |  | B | *SE* | *df* | *t* |  | B | *SE* | *df* | *t* |
| Constant | 2.49 | 0.04 | 47 | 66.01^***^ |  | 4.78 | 1.28 | 26 | 3.73^***^ |  | 0.58 | 0.08 | 47 | 7.18^***^ |  | 4.17 | 2.51 | 26 | 1.66 |  | 0.35 | 0.48 | 47 | 0.73 |  | 39.63 | 16.20 | 26 | 2.45^*^ |
| Subjective-cultural individualism-collectivism index (reversed) | 0.0002 | 0.0001 | 47 | 2.75^**^ |  | 0.00003 | 0.0001 | 26 | 0.21 |  | 0.001 | 0.0002 | 47 | 2.89^**^ |  | 0.00003 | 0.0003 | 26 | 0.12 |  | 0.004 | 0.001 | 47 | 4.17^***^ |  | 0.0001 | 0.002 | 26 | 0.07 |
| U.S. collectivism index | –0.0001 | 0.001 | 47 | –0.20 |  | –0.003 | 0.001 | 26 | –3.31^**^ |  | –0.001 | 0.002 | 47 | –1.07 |  | –0.01 | 0.002 | 26 | –5.84^***^ |  | –0.01 | 0.01 | 47 | –0.70 |  | –0.06 | 0.01 | 26 | –6.27^***^ |
| Stay–at–home index |  |  |  |  |  | 0.002 | 0.001 | 26 | 1.84 |  |  |  |  |  |  | 0.0004 | 0.002 | 26 | 0.20 |  |  |  |  |  |  | 0.02 | 0.01 | 26 | 1.79 |
| Stringency index |  |  |  |  |  | –0.002 | 0.002 | 26 | –0.93 |  |  |  |  |  |  | –0.01 | 0.004 | 26 | –2.23^*^ |  |  |  |  |  |  | –0.05 | 0.02 | 26 | –2.10^*^ |
| %COVID–19 tested |  |  |  |  |  | 0.03 | 0.003 | 26 | 10.26^***^ |  |  |  |  |  |  | 0.01 | 0.01 | 26 | 2.61^*^ |  |  |  |  |  |  | 0.29 | 0.04 | 26 | 8.01^***^ |
| %Mask wearing |  |  |  |  |  | 0.000001 | 0.002 | 26 | 0.001 |  |  |  |  |  |  | –0.003 | 0.005 | 26 | –0.67 |  |  |  |  |  |  | –0.02 | 0.03 | 26 | –0.70 |
| %Trust the CDC information |  |  |  |  |  | 0.001 | 0.004 | 26 | 0.36 |  |  |  |  |  |  | –0.01 | 0.01 | 26 | –0.68 |  |  |  |  |  |  | 0.005 | 0.05 | 26 | 0.10 |
| %Feeling anxious |  |  |  |  |  | –0.004 | 0.01 | 26 | –0.63 |  |  |  |  |  |  | 0.02 | 0.01 | 26 | 1.30 |  |  |  |  |  |  | 0.01 | 0.08 | 26 | 0.14 |
| Latitude |  |  |  |  |  | 0.0003 | 0.0004 | 26 | 0.91 |  |  |  |  |  |  | –0.001 | 0.001 | 26 | –1.20 |  |  |  |  |  |  | –0.0005 | 0.005 | 26 | –0.10 |
| Parasite–stress index |  |  |  |  |  | 0.004 | 0.002 | 26 | 2.29^*^ |  |  |  |  |  |  | 0.01 | 0.005 | 26 | 2.72^*^ |  |  |  |  |  |  | 0.11 | 0.02 | 26 | 4.50^***^ |
| HIV infection rate |  |  |  |  |  | –0.01 | 0.003 | 26 | –2.84^**^ |  |  |  |  |  |  | –0.01 | 0.01 | 26 | –1.90 |  |  |  |  |  |  | –0.14 | 0.04 | 26 | –3.63^**^ |
| Life expectancy |  |  |  |  |  | –0.002 | 0.01 | 26 | –0.21 |  |  |  |  |  |  | –0.01 | 0.02 | 26 | –0.49 |  |  |  |  |  |  | –0.02 | 0.10 | 26 | –0.22 |
| Personal income |  |  |  |  |  | –0.54 | 0.19 | 26 | –2.94^**^ |  |  |  |  |  |  | 0.04 | 0.39 | 26 | 0.09 |  |  |  |  |  |  | –5.93 | 2.34 | 26 | –2.53^*^ |
| Population density |  |  |  |  |  | 0.04 | 0.02 | 26 | 2.07^*^ |  |  |  |  |  |  | 0.13 | 0.03 | 26 | 3.79^***^ |  |  |  |  |  |  | 0.78 | 0.23 | 26 | 3.43^**^ |
| Median age |  |  |  |  |  | –0.01 | 0.004 | 26 | –1.32 |  |  |  |  |  |  | 0.01 | 0.01 | 26 | 0.92 |  |  |  |  |  |  | –0.04 | 0.05 | 26 | –0.76 |
| %Male population |  |  |  |  |  | 1.07 | 1.59 | 26 | 0.67 |  |  |  |  |  |  | –5.29 | 2.87 | 26 | –1.84 |  |  |  |  |  |  | –8.88 | 20.14 | 26 | –0.44 |
| %College degree population |  |  |  |  |  | –0.50 | 0.27 | 26 | –1.89 |  |  |  |  |  |  | –1.35 | 0.51 | 26 | –2.66^*^ |  |  |  |  |  |  | –9.95 | 3.37 | 26 | –2.95^**^ |
| Social vulnerability index |  |  |  |  |  | –0.03 | 0.07 | 26 | –0.49 |  |  |  |  |  |  | 0.04 | 0.13 | 26 | 0.28 |  |  |  |  |  |  | –0.28 | 0.84 | 26 | –0.34 |
| Airport traffic |  |  |  |  |  | 0.65 | 0.57 | 26 | 1.14 |  |  |  |  |  |  | 0.05 | 1.13 | 26 | 0.04 |  |  |  |  |  |  | 6.30 | 7.20 | 26 | 0.88 |
| %Non–white population |  |  |  |  |  | 0.003 | 0.09 | 26 | 0.03 |  |  |  |  |  |  | 0.48 | 0.18 | 26 | 2.63^*^ |  |  |  |  |  |  | 1.44 | 1.11 | 26 | 1.30 |
| Partisanship |  |  |  |  |  | 0.36 | 0.09 | 26 | 4.05^***^ |  |  |  |  |  |  | 0.38 | 0.17 | 26 | 2.20^*^ |  |  |  |  |  |  | 4.57 | 1.13 | 26 | 4.06^***^ |
| Religiosity index |  |  |  |  |  | 0.002 | 0.001 | 26 | 2.97^**^ |  |  |  |  |  |  | 0.003 | 0.002 | 26 | 1.66 |  |  |  |  |  |  | 0.03 | 0.01 | 26 | 3.31^**^ |
| Cultural tightness–looseness index |  |  |  |  |  | –0.001 | 0.001 | 26 | –1.01 |  |  |  |  |  |  | –0.003 | 0.002 | 26 | –1.85 |  |  |  |  |  |  | –0.02 | 0.01 | 26 | –1.95 |

*Note*. ^*^*p* < .05, ^**^*p* < .01, ^***^*p* < .001.

Table S3. Multilevel analysis results in Study 2

| Predictor | Outcome variable | | | | | | | | | | | | | | | | | | | | | | | | | | | | |
| --- | --- | --- | --- | --- | --- | --- | --- | --- | --- | --- | --- | --- | --- | --- | --- | --- | --- | --- | --- | --- | --- | --- | --- | --- | --- | --- | --- | --- | --- |
|  | COVID–19 cases per million | | | | | | | | |  | COVID–19 deaths per million | | | | | | | | |  | Composite COVID–19 severity index | | | | | | | | |
|  | Model 1 without covariates  ICC = .377 | | | |  | Model 2 with covariates  ICC = .333 | | | |  | Model 1 without covariates  ICC = .349 | | | |  | Model 2 with covariates  ICC = .430 | | | |  | Model 1 without covariates  ICC = .418 | | | |  | Model 2 with covariates  ICC = .375 | | | |
|  | B | *SE* | *df* | *t* |  | B | *SE* | *df* | *t* |  | B | *SE* | *df* | *t* |  | B | *SE* | *df* | *t* |  | B | *SE* | *df* | *t* |  | B | *SE* | *df* | *t* |
| Constant | 2.53 | 0.04 | 3129 | 58.60^***^ |  | 2.76 | 0.24 | 2088 | 11.68^***^ |  | 0.68 | 0.08 | 3117 | 8.37^***^ |  | 4.25 | 0.42 | 2088 | 10.00^***^ |  | 0.85 | 0.38 | 3129 | 2.24^*^ |  | 10.70 | 1.84 | 2088 | 5.81^***^ |
| State-Level Subjective-cultural individualism-collectivism index (reversed) | 0.0004 | 0.0001 | 3129 | 3.84^***^ |  | 0.0003 | 0.0022 | 2088 | 1.81 |  | 0.001 | 0.0002 | 3117 | 6.25^*^^**^ |  | –0.0002 | 0.0003 | 2088 | –0.72 |  | 0.005 | 0.001 | 3129 | 5.97^***^ |  | 0.001 | 0.001 | 2088 | 0.71 |
| State-Level U.S. collectivism index | –0.001 | 0.001 | 3129 | –1.75 |  | –0.002 | 0.001 | 2088 | –2.08^*^ |  | –0.003 | 0.002 | 3117 | –1.78 |  | –0.01 | 0.002 | 2088 | –2.59^*^ |  | –0.02 | 0.01 | 3129 | –2.91^*^ |  | –0.03 | 0.01 | 2088 | –2.85^**^ |
| County–level stay–at–home index |  |  |  |  |  | –0.001 | 0.0001 | 2088 | –3.59^***^ |  |  |  |  |  |  | 0.0001 | 0.0002 | 2088 | 0.50 |  |  |  |  |  |  | –0.002 | 0.001 | 2088 | –2.26^*^ |
| County–level latitude |  |  |  |  |  | –0.004 | 0.001 | 2088 | –4.10^***^ |  |  |  |  |  |  | 0.002 | 0.002 | 2088 | 1.07 |  |  |  |  |  |  | –0.02 | 0.01 | 2088 | –2.26^*^ |
| County–level parasite–stress index |  |  |  |  |  | –0.001 | 0.004 | 2088 | –0.30 |  |  |  |  |  |  | 0.02 | 0.01 | 2088 | 2.99^**^ |  |  |  |  |  |  | 0.05 | 0.03 | 2088 | 1.46 |
| County–level HIV infection rate |  |  |  |  |  | –0.00004 | 0.00001 | 2088 | –3.16^**^ |  |  |  |  |  |  | –0.00008 | 0.00002 | 2088 | –4.18^***^ |  |  |  |  |  |  | –0.0004 | 0.0001 | 2088 | –4.52^***^ |
| County–level life expectancy |  |  |  |  |  | –0.004 | 0.001 | 2088 | –4.19^***^ |  |  |  |  |  |  | –0.02 | 0.002 | 2088 | –13.75^***^ |  |  |  |  |  |  | –0.07 | 0.01 | 2088 | –10.61^***^ |
| County–level personal income |  |  |  |  |  | 0.06 | 0.02 | 2088 | 2.36^*^ |  |  |  |  |  |  | –0.30 | 0.038 | 2088 | –7.93^***^ |  |  |  |  |  |  | –0.48 | 0.17 | 2088 | –2.77^**^ |
| County–level population density |  |  |  |  |  | 0.003 | 0.004 | 2088 | 0.67 |  |  |  |  |  |  | –0.02 | 0.006 | 2088 | –3.24^**^ |  |  |  |  |  |  | –0.04 | 0.03 | 2088 | –1.32 |
| County–level %male population |  |  |  |  |  | 0.005 | 0.001 | 2088 | 5.89^***^ |  |  |  |  |  |  | –0.002 | 0.001 | 2088 | –1.81 |  |  |  |  |  |  | 0.02 | 0.01 | 2088 | 3.08^**^ |
| County–level %college degree population |  |  |  |  |  | –0.0004 | 0.0004 | 2088 | –0.88 |  |  |  |  |  |  | 0.000 | 0.001 | 2088 | –0.28 |  |  |  |  |  |  | –0.003 | 0.003 | 2088 | –0.83 |
| County–level median age |  |  |  |  |  | –0.004 | 0.003 | 2088 | –12.11^***^ |  |  |  |  |  |  | 0.01 | 0.001 | 2088 | 14.66^***^ |  |  |  |  |  |  | –0.001 | 0.003 | 2088 | –0.26 |
| County–level social vulnerability index |  |  |  |  |  | 0.03 | 0.01 | 2088 | 3.61^***^ |  |  |  |  |  |  | 0.12 | 0.01 | 2088 | 8.02^***^ |  |  |  |  |  |  | 0.49 | 0.07 | 2088 | 6.96^***^ |
| County–level partisanship |  |  |  |  |  | 0.03 | 0.01 | 2088 | 2.94^**^ |  |  |  |  |  |  | 0.10 | 0.01 | 2088 | 7.17^***^ |  |  |  |  |  |  | 0.38 | 0.06 | 2088 | 6.06^***^ |
| County–level religiosity index |  |  |  |  |  | 0.00008 | 0.00001 | 2088 | 6.87^***^ |  |  |  |  |  |  | 0.0002 | 0.00002 | 2088 | 13.59^***^ |  |  |  |  |  |  | 0.001 | 0.0001 | 2088 | 12.33^***^ |
| State–level stringency index |  |  |  |  |  | –0.0001 | 0.003 | 2088 | –0.04 |  |  |  |  |  |  | –0.01 | 0.005 | 2088 | –1.37 |  |  |  |  |  |  | –0.02 | 0.02 | 2088 | –0.80 |
| State–level %COVID–19 tested |  |  |  |  |  | 0.01 | 0.004 | 2088 | 2.75^**^ |  |  |  |  |  |  | 0.003 | 0.01 | 2088 | 0.39 |  |  |  |  |  |  | 0.06 | 0.03 | 2088 | 1.95 |
| State–level %mask wearing |  |  |  |  |  | 0.004 | 0.003 | 2088 | 1.54 |  |  |  |  |  |  | 0.01 | 0.005 | 2088 | 1.75 |  |  |  |  |  |  | 0.04 | 0.02 | 2088 | 2.00^*^ |
| State–level %trust the CDC information |  |  |  |  |  | –0.004 | 0.003 | 2088 | –1.37 |  |  |  |  |  |  | –0.02 | 0.01 | 2088 | –3.20^**^ |  |  |  |  |  |  | –0.07 | 0.03 | 2088 | –2.75^**^ |
| State–level %feeling anxious |  |  |  |  |  | –0.01 | 0.01 | 2088 | –1.85 |  |  |  |  |  |  | –0.02 | 0.01 | 2088 | –1.59 |  |  |  |  |  |  | –0.12 | 0.05 | 2088 | –2.14^*^ |
| State–level %non–white population |  |  |  |  |  | –0.14 | 0.07 | 2088 | –2.09^*^ |  |  |  |  |  |  | 0.22 | 0.13 | 2088 | 1.68 |  |  |  |  |  |  | –0.17 | 0.56 | 2088 | –0.31 |
| State–level airport traffic |  |  |  |  |  | –0.04 | 0.57 | 2088 | –0.07 |  |  |  |  |  |  | 1.32 | 1.09 | 2088 | 1.21 |  |  |  |  |  |  | 2.99 | 4.63 | 2088 | 0.65 |
| State–level cultural tightness–looseness index |  |  |  |  |  | –0.001 | 0.001 | 2088 | –1.10 |  |  |  |  |  |  | 0.000 | 0.002 | 2088 | –0.16 |  |  |  |  |  |  | –0.01 | 0.01 | 2088 | –0.78 |

*Note*. ^*^*p* < .05, ^**^*p* < .01, ^***^*p* < .001.

Table S4. Multilevel analysis results in Study 3

| Predictor | Outcome variable | | | | | | | | | | | | | | | | | | | | | | | | | | | |
| --- | --- | --- | --- | --- | --- | --- | --- | --- | --- | --- | --- | --- | --- | --- | --- | --- | --- | --- | --- | --- | --- | --- | --- | --- | --- | --- | --- | --- |
|  | COVID–19 cases per million | | | | | | | | |  | COVID–19 deaths per million | | | | | | | | | Composite COVID–19 Severity Index | | | | | | | | |
|  | Model 1 without covariates  ICC = .377 | | | |  | Model 2 with covariates  ICC = .333 | | | |  | Model 1 without covariates  ICC = .349 | | | |  | Model 2 with covariates  ICC = .430 | | | | Model 1 without covariates  ICC = .349 | | | |  | Model 2 with covariates  ICC = .430 | | | |
|  | B | *SE* | *df* | *t* |  | B | *SE* | *df* | *t* |  | B | *SE* | *df* | *t* |  | B | *SE* | *df* | *t* | B | *SE* | *df* | *t* |  | B | *SE* | *df* | *t* |
| Constant | 2.27 | 0.07 | 40414 | 30.39^***^ |  | 1.29 | 1.11 | 35198 | 1.16 |  | 0.63 | 0.09 | 32868 | 6.74^***^ |  | 2.10 | 1.96 | 25765 | 1.07 | 0.05 | 0.13 | 40675 | 0.42 |  | 0.04 | 2.48 | 35458 | 0.02 |
| State-Level Subjective-cultural individualism-collectivism index (reversed) | 0.0004 | 0.0002 | 40414 | 2.27^*^ |  | –0.000003 | 0.0001 | 35198 | –0.02 |  | 0.001 | 0.0002 | 32868 | 2.54^*^ |  | –0.0002 | 0.0002 | 25765 | –0.78 | 0.001 | 0.0003 | 40675 | 2.74^**^ |  | –0.0001 | 0.0003 | 35458 | –0.50 |
| State-Level U.S. collectivism index | –0.001 | 0.001 | 40414 | –0.61 |  | –0.002 | 0.001 | 35198 | –3.10^**^ |  | –0.003 | 0.002 | 32868 | –1.92 |  | –0.003 | 0.001 | 25765 | –2.35^*^ | –0.002 | 0.003 | 40675 | –0.87 |  | –0.005 | 0.002 | 35458 | –3.18^**^ |
| Daily–level preceeding–day COVID–19 epidemiological data |  |  |  |  |  | 0.66 | 0.004 | 35198 | 170.85^***^ |  |  |  |  |  |  | 0.46 | 0.01 | 25765 | 85.46^***^ |  |  |  |  |  | 0.57 | 0.004 | 35458 | 134.65^***^ |
| Daily–level day |  |  |  |  |  | 0.0004 | 0.00002 | 35198 | 21.32^***^ |  |  |  |  |  |  | 0.0002 | 0.00002 | 25765 | 7.01^***^ |  |  |  |  |  | 0.001 | 0.00003 | 35458 | 26.34^***^ |
| Daily–level season |  |  |  |  |  | 0.15 | 0.005 | 35198 | 33.21^***^ |  |  |  |  |  |  | 0.14 | 0.01 | 25765 | 25.37^***^ |  |  |  |  |  | 0.28 | 0.01 | 35458 | 36.39^***^ |
| Daily–level stay–at–home index |  |  |  |  |  | 0.002 | 0.0001 | 35198 | 16.81^***^ |  |  |  |  |  |  | 0.004 | 0.0001 | 25765 | 25.79^***^ |  |  |  |  |  | 0.004 | 0.0002 | 35458 | 22.22^***^ |
| Daily–level stringency index |  |  |  |  |  | 0.002 | 0.003 | 35198 | 8.08^***^ |  |  |  |  |  |  | 0.001 | 0.0003 | 25765 | 4.29^***^ |  |  |  |  |  | 0.01 | 0.0004 | 35458 | 14.17^***^ |
| State–level %COVID–19 tested |  |  |  |  |  | 0.005 | 0.003 | 35198 | 1.83 |  |  |  |  |  |  | 0.01 | 0.005 | 25765 | 2.20^*^ |  |  |  |  |  | 0.01 | 0.01 | 35458 | 1.59 |
| State–level %mask wearing |  |  |  |  |  | –0.002 | 0.002 | 35198 | –1.13 |  |  |  |  |  |  | –0.01 | 0.01 | 25765 | –2.29^*^ |  |  |  |  |  | –0.01 | 0.004 | 35458 | –1.47 |
| State–level %trust the CDC information |  |  |  |  |  | 0.01 | 0.003 | 35198 | 2.15^*^ |  |  |  |  |  |  | 0.01 | 0.01 | 25765 | 1.59 |  |  |  |  |  | 0.01 | 0.01 | 35458 | 1.90 |
| State–level %feeling anxious |  |  |  |  |  | –0.01 | 0.01 | 35198 | –2.83^**^ |  |  |  |  |  |  | –0.01 | 0.01 | 25765 | –1.12 |  |  |  |  |  | –0.03 | 0.01 | 35458 | –2.43^*^ |
| State–level latitude |  |  |  |  |  | 0.0003 | 0.0003 | 35198 | 0.88 |  |  |  |  |  |  | 0.0004 | 0.001 | 25765 | 0.63 |  |  |  |  |  | 0.0005 | 0.001 | 35458 | 0.70 |
| State–level parasite–stress index |  |  |  |  |  | 0.005 | 0.002 | 35198 | 2.81^**^ |  |  |  |  |  |  | 0.003 | 0.003 | 25765 | 0.91 |  |  |  |  |  | 0.01 | 0.004 | 35458 | 1.38 |
| State–level HIV infection rate |  |  |  |  |  | 0.002 | 0.002 | 35198 | 0.71 |  |  |  |  |  |  | –0.0004 | 0.004 | 25765 | –0.11 |  |  |  |  |  | 0.01 | 0.005 | 35458 | 1.43 |
| State–level life expectancy |  |  |  |  |  | –0.003 | 0.01 | 35198 | –0.44 |  |  |  |  |  |  | –0.02 | 0.01 | 25765 | –1.34 |  |  |  |  |  | –0.02 | 0.01 | 35458 | –1.04 |
| State–level personal income |  |  |  |  |  | –0.15 | 0.17 | 35198 | –0.87 |  |  |  |  |  |  | 0.06 | 0.30 | 25765 | 0.19 |  |  |  |  |  | 0.18 | 0.39 | 35458 | 0.47 |
| State–level population density |  |  |  |  |  | 0.03 | 0.02 | 35198 | 1.98^*^ |  |  |  |  |  |  | 0.03 | 0.03 | 25765 | 1.14 |  |  |  |  |  | 0.10 | 0.04 | 35458 | 2.83^**^ |
| State–level median age |  |  |  |  |  | –0.01 | 0.003 | 35198 | –3.02^**^ |  |  |  |  |  |  | –0.01 | 0.01 | 25765 | –1.04 |  |  |  |  |  | –0.02 | 0.01 | 35458 | –2.65^**^ |
| State–level %male population |  |  |  |  |  | 1.32 | 1.46 | 35198 | 0.90 |  |  |  |  |  |  | –0.41 | 2.51 | 25765 | –0.16 |  |  |  |  |  | 2.00 | 3.27 | 35458 | 0.61 |
| State–level %college degree population |  |  |  |  |  | –0.52 | 0.24 | 35198 | –2.18^*^ |  |  |  |  |  |  | –0.85 | 0.40 | 25765 | –2.10^*^ |  |  |  |  |  | –1.53 | 0.53 | 35458 | –2.88^**^ |
| State–level social vulnerability index |  |  |  |  |  | 0.03 | 0.06 | 35198 | 0.43 |  |  |  |  |  |  | 0.03 | 0.10 | 25765 | 0.31 |  |  |  |  |  | 0.10 | 0.14 | 35458 | 0.72 |
| State–level airport traffic |  |  |  |  |  | –0.63 | 0.52 | 35198 | –1.21 |  |  |  |  |  |  | –2.51 | 0.89 | 25765 | –2.83^**^ |  |  |  |  |  | –3.58 | 1.17 | 35458 | –3.05^**^ |
| State–level %non–white population |  |  |  |  |  | –0.22 | 0.06 | 35198 | –3.46^***^ |  |  |  |  |  |  | –0.22 | 0.11 | 25765 | –1.92 |  |  |  |  |  | –0.42 | 0.14 | 35458 | –2.95^**^ |
| State–level partisanship |  |  |  |  |  | 0.16 | 0.08 | 35198 | 2.02^*^ |  |  |  |  |  |  | 0.10 | 0.14 | 25765 | 0.73 |  |  |  |  |  | 0.32 | 0.18 | 35458 | 1.83 |
| State–level religiosity index |  |  |  |  |  | 0.000004 | 0.001 | 35198 | 0.01 |  |  |  |  |  |  | 0.002 | 0.001 | 25765 | 1.41 |  |  |  |  |  | 0.0001 | 0.002 | 35458 | 0.07 |
| State–level cultural tightness–looseness index |  |  |  |  |  | 0.0004 | 0.001 | 35198 | 0.44 |  |  |  |  |  |  | –0.001 | 0.002 | 25765 | –0.37 |  |  |  |  |  | 0.001 | 0.002 | 35458 | 0.41 |

*Note*. ^*^*p* < .05, ^**^*p* < .01, ^***^*p* < .001.

Appendix 1. Data sources and timeframes for variables included in all studies

| Study | Level | Variable | Timeframe | Data Source |
| --- | --- | --- | --- | --- |
| Study 1 | State | COVID-19 cases and deaths per million | the earliest date when the first COVID-19 confirmed cases were documented to December 31 2022 | https://github.com/nytimes |
| Study 1 | State | State-Level Subjective-cultural individualism-collectivism index (reversed) | Various years | JA Vandello and D Cohen [1] |
| Study 1 | State | State-Level U.S. collectivism index | Various years | M Minkov and A Kaasa [2] |
| Study 1 | State | Stay-at-home index | 15 February 2020 to 15 October 2022 | https://www.google.com/covid19/mobility/ |
| Study 1 | State | Stringency index | 1 January 2020 to 31 December 2022 | https://www.bsg.ox.ac.uk/research/research-projects/covid-19-government-response-tracker |
| Study 1 | State | %people who were tested for COVID-19 in the past 14 days (regardless of their test result), | 8 September 2020 to 15 March 2021 | https://delphi.cmu.edu/ |
| Study 1 | State | %people wearing mask in last 5 days | 8 September 2020 to 15 March 2021 | https://delphi.cmu.edu/ |
| Study 1 | State | %people who trust the Centers for Disease Control (CDC) to provide accurate news and information about COVID-19 | 8 September 2020 to 15 March 2021 | https://delphi.cmu.edu/ |
| Study 1 | State | %people reporting feeling anxious about the virus for the past 5 days | 8 September 2020 to 15 March 2021 | https://delphi.cmu.edu/ |
| Study 1 | State | Latitude | NA | https://developers.google.com/public-data |
| Study 1 | State | Historical parasite-stress index | 1979 to 1998 | https://wonder.cdc.gov/ |
| Study 1 | State | HIV infection rate | 2020 | https://www.statista.com/statistics/257734/us-states-with-highest-aids-diagnosis-rates/ |
| Study 1 | State | Life expectancy | 2020 | https://www.countyhealthrankings.org/ |
| Study 1 | State | Personal income | 2019 | https://www.bea.gov/data/income-saving/personal-income-by-state |
| Study 1 | State | Population density | 2010 | https://www.census.gov/quickfacts/geo/chart/US/POP060210 |
| Study 1 | State | Median age | 2019 | https://www.statista.com/statistics/208048/median-age-of-population-in-the-usa-by-state/ |
| Study 1 | State | %male population | 2019 | https://www.kff.org/ |
| Study 1 | State | Education level | 2015 to 2019 | https://data.ers.usda.gov/reports.aspx?ID=17829 |
| Study 1 | State | Social vulnerability index | 2020 | https://www.atsdr.cdc.gov/placeandhealth/svi/data_documentation_download.html |
| Study 1 | State | Airport traffic | Various years | <https://github>.com/satunr/COVID-19/tree/master/US-COVID-Dataset |
| Study 1 | State | %non-white population | 2019 | <https://www>.kff.org/ |
| Study 1 | State | Political affiliation | 2016 | MZ Ma and S Ye [3] |
| Study 1 | State | Religiosity index | 2004 to 2020 | MZ Ma and S Ye [3] |
| Study 1 | State | Cultural tightness-looseness index | Various years | AS Wormley and AB Cohen [4] |
| Study 2 | County | Stay-at-home index | 15 February 2020 to 15 October 2022 | https://www.google.com/covid19/mobility/ |
| Study 2 | County | COVID-19 epidemiological data | the earliest date when the first COVID-19 confirmed cases were documented to December 31 2022 | https://github.com/nytimes |
| Study 2 | County | Latitude | NA | MZ Ma and S Ye [3] |
| Study 2 | County | Parasite-stress index | 1980 to 2014 | MZ Ma and S Ye [3] |
| Study 2 | County | HIV infection rate | 2020 | https://www.countyhealthrankings.org/ |
| Study 2 | County | Personal income | 2019 | https://www.bea.gov/data/income-saving/personal-income-county-metro-and-other-areas |
| Study 2 | County | Population density | 2010 | https://www.census.gov/data/datasets/time-series/demo/popest/2010s-counties-total.html |
| Study 2 | County | %male population | 2010 to 2019 | https://www2.census.gov/programs-surveys/popest/datasets/2010-2019/counties/asrh/ |
| Study 2 | County | Education level | 1970 to 2021 | https://www.ers.usda.gov/data-products/county-level-data-sets/download-data |
| Study 2 | County | Median age | 2010 to 2019 | https://www2.census.gov/programs-surveys/popest/datasets/2010-2019/counties/asrh/ |
| Study 2 | County | Social vulnerability index | 2020 | https://www.atsdr.cdc.gov/placeandhealth/svi/data_documentation_download.html |
| Study 2 | County | Political affiliation | 2016 | MZ Ma and S Ye [3] |
| Study 2 | County | Religiosity index | 2010 | https://www.thearda.com/data-archive/browse-categories |
| Study 3 | Daily | COVID-19 epidemiological data | the earliest date when the first COVID-19 confirmed cases were documented to December 31 2022 | https://github.com/nytimes |
| Study 3 | Daily | Stay-at-home index | 15 February 2020 to 15 October 2022 | https://www.google.com/covid19/mobility/ |
| Study 3 | Daily | Stringency index | 1 January 2020 to 31 December 2022 | https://www.bsg.ox.ac.uk/research/research-projects/covid-19-government-response-tracker |

**References**

1. Vandello JA, Cohen D: **Patterns of individualism and collectivism across the United States**. *Journal of Personality and Social Psychology* 1999, **77**(2):279-292.

2. Minkov M, Kaasa A: **A test of the revised Minkov-Hofstede model of culture: mirror images of subjective and objective culture across nations and the 50 US states**. *Cross-Cultural Research* 2021, **55**(2-3):230-281.

3. Ma MZ, Ye S: **The role of ingroup assortative sociality in the COVID-19 pandemic: A multilevel analysis of google trends data in the United States**. *International Journal of Intercultural Relations* 2021, **84**:168-180.

4. Wormley AS, Cohen AB: **CHEAT: Wordle Cheating Is Related to Religiosity and Cultural Tightness**. *Perspectives on Psychological Science* 2023, **18**(3):702-709.
